# Full-length single-cell RNA-seq applied to a viral human cancer: applications to HPV expression and splicing analysis in HeLa S3 cells

**DOI:** 10.1186/s13742-015-0091-4

**Published:** 2015-11-05

**Authors:** Liang Wu, Xiaolong Zhang, Zhikun Zhao, Ling Wang, Bo Li, Guibo Li, Michael Dean, Qichao Yu, Yanhui Wang, Xinxin Lin, Weijian Rao, Zhanlong Mei, Yang Li, Runze Jiang, Huan Yang, Fuqiang Li, Guoyun Xie, Liqin Xu, Kui Wu, Jie Zhang, Jianghao Chen, Ting Wang, Karsten Kristiansen, Xiuqing Zhang, Yingrui Li, Huanming Yang, Jian Wang, Yong Hou, Xun Xu

**Affiliations:** 1 BGI-Shenzhen, Shenzhen, 518083 China; 2 College of Life Sciences, University of Chinese Academy of Sciences, Beijing, 100049 China; 3 State Key Laboratory of Bioelectronics, Southeast University, Nanjing, 210096 China; 4 School of Biological Science and Medical Engineering, Southeast University, Nanjing, 210096 China; 5 Department of Vascular and Endocrine Surgery, Xijing Hospital, Fourth Military Medical University, Xi’an, 710032 China; 6 Department of Biology, University of Copenhagen, Copenhagen, 1599 Denmark; 7 Cancer and Inflammation Program, National Cancer Institute at Frederick, Building 560, Frederick, MD 21702 USA; 8 BGI-Education Center, University of Chinese Academy of Sciences, Shenzhen, 518083 China; 9 The Guangdong Enterprise Key Laboratory of Human Disease Genomics, BGI-Shenzhen, Shenzhen, 518083 China; 10 Institute for Molecular Bioscience, The University of Queensland, Brisbane, QLD 4072 Australia; 11 James D. Watson Institute of Genome Sciences, Zhejiang University, Hangzhou, 310058 China

**Keywords:** Single-cell transcriptome, HeLa, HPV, Virus, Tumor heterogeneity, Cancer, RNA splicing

## Abstract

**Background:**

Viral infection causes multiple forms of human cancer, and HPV infection is the primary factor in cervical carcinomas. Recent single-cell RNA-seq studies highlight the tumor heterogeneity present in most cancers, but virally induced tumors have not been studied. HeLa is a well characterized HPV+ cervical cancer cell line.

**Result:**

We developed a new high throughput platform to prepare single-cell RNA on a nanoliter scale based on a customized microwell chip. Using this method, we successfully amplified full-length transcripts of 669 single HeLa S3 cells and 40 of them were randomly selected to perform single-cell RNA sequencing. Based on these data, we obtained a comprehensive understanding of the heterogeneity of HeLa S3 cells in gene expression, alternative splicing and fusions. Furthermore, we identified a high diversity of HPV-18 expression and splicing at the single-cell level. By co-expression analysis we identified 283 E6, E7 co-regulated genes, including *CDC25*, *PCNA*, *PLK4*, *BUB1B* and *IRF1* known to interact with HPV viral proteins.

**Conclusion:**

Our results reveal the heterogeneity of a virus-infected cell line. It not only provides a transcriptome characterization of HeLa S3 cells at the single cell level, but is a demonstration of the power of single cell RNA-seq analysis of virally infected cells and cancers.

**Electronic supplementary material:**

The online version of this article (doi:10.1186/s13742-015-0091-4) contains supplementary material, which is available to authorized users.

## Background

Virus infection causes approximately 12 % of cancers in the world [1–4]. Human papilloma virus (HPV), Epstein-Barr virus (EBV), hepatitis B virus (HBV), Kaposi’s sarcoma-associated herpes virus (KSHV), Merkel cell polyomavirus (MCPyV), hepatitis C virus (HCV), Human immunodeficiency virus (HIV) and human T cell lymphotropic virus type 1 (HTLV-1) are associated with multiple forms of malignancies [4–11]. In particular, nearly all cervical cancers are caused by high risk HPV infections [12]. The underlying mechanisms of virus-triggered cellular changes are signaling mimicry, effects on the DNA damage response, virally encoded oncogenes and chronic inflammatory responses to persistent viral infection [4, 13–16]. Tumor heterogeneity creates a challenge in the development of cancer treatments [17–20]. Recent single-cell RNA-seq techniques have been used to investigate the inter/intra-tumor heterogeneity in gene expression, alternative splicing variants and SNVs [21–24]. However, there has not been any investigation of the heterogeneity of virally infected tumors by single-cell RNA-seq.

The HeLa cell line is the most widely used model in biology research, and is a virus-infected cell line derived from cervical tumor tissue established in 1951 [25]. Recently whole genome and transcriptome sequencing of different HeLa strains were used to comprehensively understand the HeLa cell line [26, 27]. These studies indicate that HeLa has a high level of aneuploidy, numerous large structural variants, extensive point mutations and extensive genomic rearrangement, especially at chromosome 8q24.21, the hotspot site HPV-18 genome integration [26, 27]. However, the HPV integrations and point mutations are relatively stable over multiple HeLa cell isolates [26, 27]. Therefore HeLa is a good object for a pilot study to investigate the tumor heterogeneity in cervical cancer and other virus-infected cancers based on single-cell transcriptome analysis.

Here, we developed a microwell full-length mRNA amplification and library construction system (MIRALCS), allowing massively parallel single-cell full-length transcripts amplification for whole transcriptome sequencing. Using this new pipeline, we sequenced single-cell transcriptomes of 40 HeLa S3 cells, and demonstrated extensive heterogeneity of this virus-infected cell line in gene expression, alternative splicing, and fusion. Furthermore, we also found a set of genes which are potential interactors with or regulated by E6, E7 based on co-expression analyses. Most interestingly, we reported a high diversity of HPV-host expression and splicing at the single-cell level.

### Date description

We collected the HeLa S3 cells by using standard cultured cell collection procedures. We carried out full-length mRNA amplification of single cells and total RNA from cell populations using both MIRALCS and traditional tube-based methods (Methods). We identified 669/4464 target wells of HeLa S3 cells prepared by MIRALCS, and randomly selected 40/669 amplified cDNA products of single cells and 5/144 replicates of 10 pg total RNA to do subsequent library preparation. Of all 45 libraries, 37 single-cell and 5 replicated 10 pg total RNA libraries were sequenced on Hiseq 2000 for single-end 49 bp length (SE50, mean 6 million reads per library), while 8 single-cell libraries (5 cells overlapped with the front cells) were sequenced on the same platform but with paired-end 150 bp length reads and much deeper sequencing (PE151, mean 27 million reads per library) for additional analysis beyond expression profiling (Additional file 1: Figure S1). External RNA Controls Consortium (ERCC) spike-in mRNAs were added in the cell lysis buffer of 19 of the 37 single cells as well as all 5 replicates of 10 pg RNAs (Additional file 1: Figure S1), and were used for the assessment of MIRALCS and absolutely quantification of the mRNA molecular counts of each library. For the tube-based method, the amplified cDNA of 5 single HeLa cells picked by mouth pipette, 3 repeats of diluted 10 pg total RNA were prepared following SMART-Seq2 protocol [28], and were sequenced into SE50 reads (mean 7 million reads per library). In addition, one 5 ng bulk RNA as a control was amplified by a tube-based approach and sequenced into both SE50 (8 million reads) and PE91 (46 million reads). All of these data were mapped to human reference sequence and with a mean mapping rate of ~75 %. Detailed sample information and sequencing data information were summarized in Additional file 1: Table S1, S2.

### Analyses

#### A new full-length RNA sequencing method (MIRALCS)

**Fig. 1:**
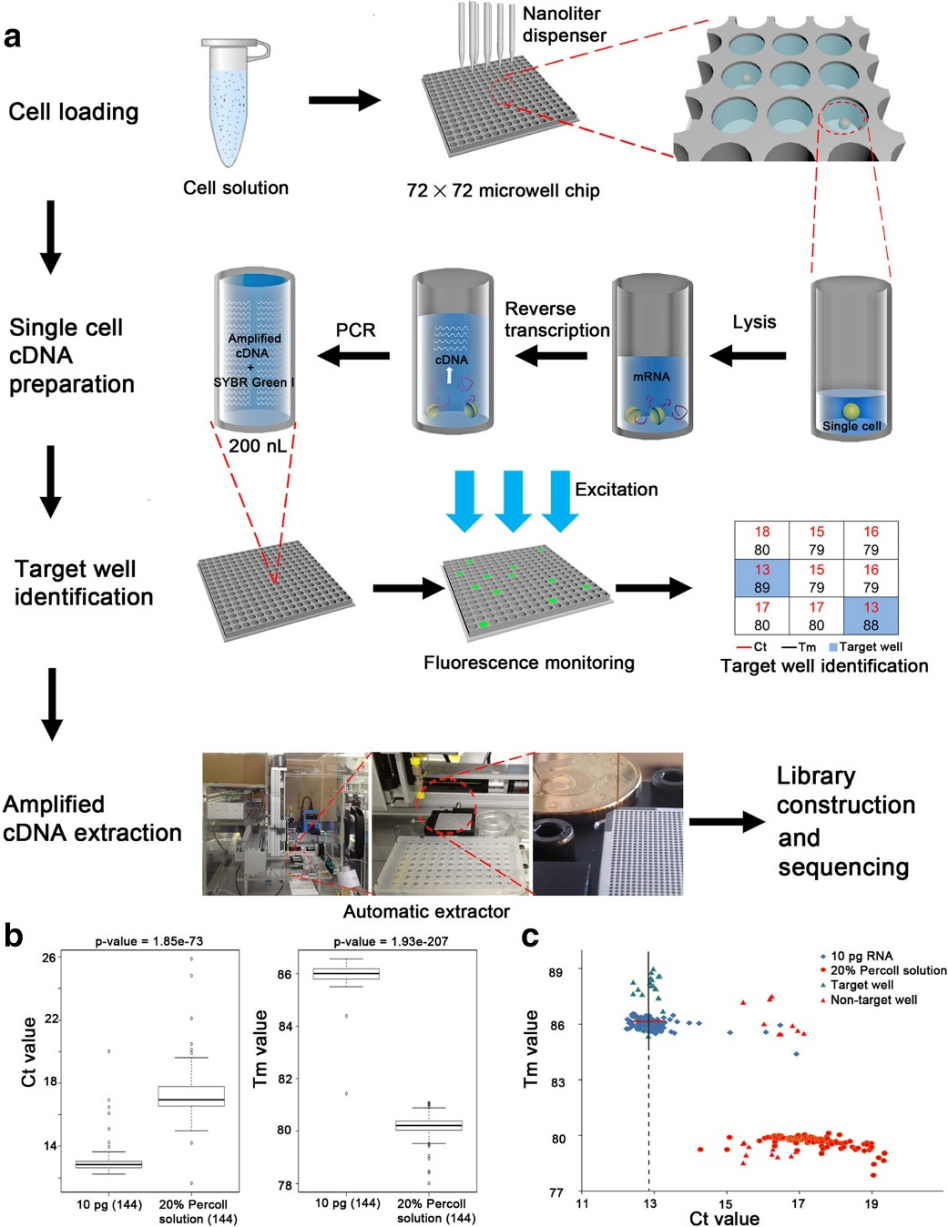
The schematic diagram of MIRALCS. **a** The flowchart of the MIRALCS. **b** The box plot of Ct (*left*) and Tm (*right*) value of the 20 % Percoll solution (negative control) and 10 pg total RNA (positive control), respectively. **c** The Ct value and Tm value distribution of 20 % Percoll solution, 10 pg total RNA, non-target well and target well during cDNA amplification process in microwells. The target wells (well with cell) and the non-target wells (without cells) were validated by Agilent 2100 Bioanlyzer. The *line* denotes Ct median. *Horizontal bars* denote ± 0.5

To improve the throughput and reduce the reagent consumption of single-cell RNA preparation, we established a new pipeline called MIRALCS. In MIRALCS, we carried out the entire process from single-cell separation to cDNA amplification in a customized 200 nl 5184-well microwell chip. The cDNA products can be transported by an automatic extractor, constructed into libraries and sequenced. The main steps of MIRALCS include cell loading, single-cell cDNA preparation, target well identification, amplified cDNA product extraction and library construction (Fig. 1a).

We dispensed the lysis buffer with RNase inhibitor into the microwells to stabilize RNA during the cell loading, and cell separation can be carried out in 15 min to reduce RNA degradation. The cell distribution follows a Poisson distribution [29]. To decrease cell sedimentation velocity, we used Percoll solution and found ~90 % of cells remaining in suspension after 30 min when cell concentration was <5 cells/μl in 20 % Percoll (Methods, Additional file 1: Table S3). To select a suitable cell concentration, we tested the cell distribution at different concentrations (Methods). We tested several cell concentrations (Additional file 1: Figure S2), and chose 2 to 8 cells/μl to balance the percentages of wells with single cell and those with multiple cells.

We followed the modified SMART-seq2 protocol [28] to complete RNA reverse transcription and cDNA amplification (Methods), to enrich for full-length transcripts in single cells. Because there are up to 5184 wells on the chip, we developed a new semi-automated method to identify positive wells. We used cycle threshold (Ct) and melting temperature (Tm) values to discriminate amplified cDNA products from primer dimers (Fig. 1b, Additional file 1: Figure S3). The Ct and Tm values showed a significant difference between negative controls and positive controls (*P* < 0.001, Fig. 1b). We used combined cutoff values of Ct median ± 0.5 and Tm > 85 to identify target wells. To test the false positive rate, we randomly extracted products (20 predicted target wells with cDNA, 20 predicted non-target wells without products, 5 wells of negative controls, and 5 wells of positive controls; Fig. 1a, c, Additional file 1: Table S4), and found no false positives or negatives (Fig. 1c, Additional file 1: Figure S4). The yields of the cDNA products from each well were 0.5 ~ 3 ng. Then we used a customized automatic extractor to transport the products of 45 wells (40 single HeLa cell and 5 replicates of 10 pg RNA wells) from microwell chip to a 96-well plate for library construction and sequencing (Methods).

### Sensitivity, accuracy and reproducibility of MIRALCS

**Fig. 2:**
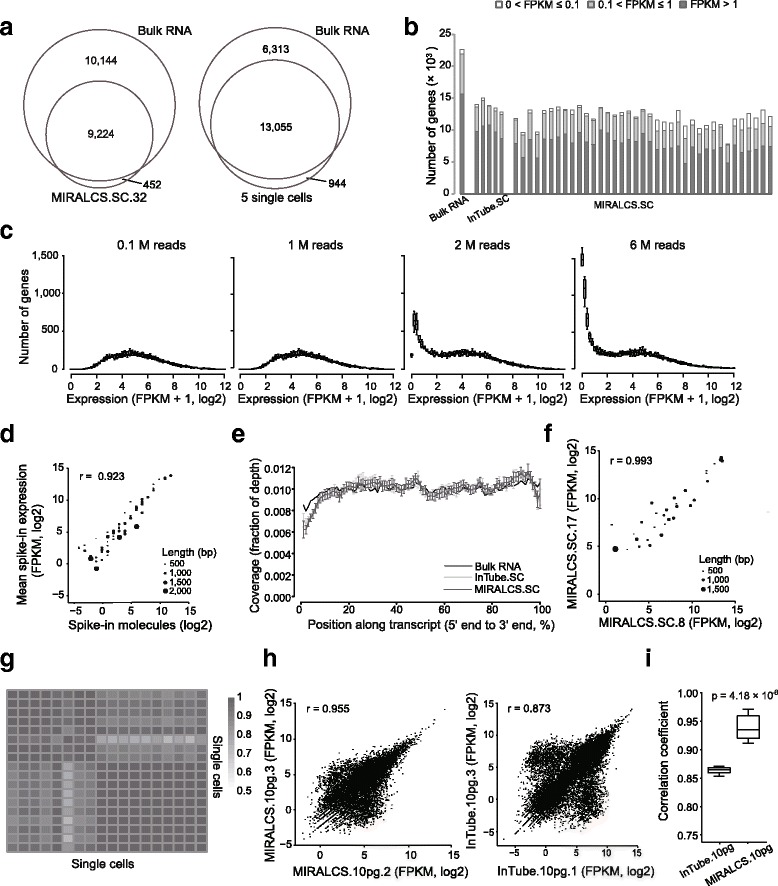
A high sensitivity, accuracy and reproducibility of MIRALCS. **a** Comparison of gene number between single cell (the *smaller circle*) and the 5 ng bulk sample (the *larger circle*). The left, a typical cell; the right, 5 randomly selected cells (randomly sampling 0.4 million reads per cell) vs. the 5 ng bulk sample (2 million reads). **b** Gene detection in MIRALCS single-cells, regular tube-based single cells and 5 ng bulk RNA sample. **c** The distribution of gene number on gene expression along sequencing depths. **d** The correlation of the mean expression (FPKM) and the number of input molecules of spike-ins of all MIRALCS single-cell libraries. **e** The reads coverage along the transcript position from 5′ to 3′end. Error bar stands for the standard deviation. **f** The correlation of spike-ins expression (FPKM) between two randomly selected MIRALCS single cells. **g** Heat map of correlation coefficients of spike-ins expression levels with input molecules >1 for each library (*n* = 19). **h** The correlation of gene expression (FPKM) between technical replicates. *Left*: two randomly selected MIRALCS 10 pg replicates. *Right*: two randomly selected tube-based 10 pg replicates. **i** The pair-wise correlation in MIRALCS 10 pg RNA replicates and tube-based 10 pg RNA replicates

To assess the sensitivity of MIRALCS, we performed a comparison of tube-based single cells and bulk RNA vs. MIRALCS single cells on gene detection. To assess the gene detection sensitivity and efficiency between MIRALCS single cells and bulk RNA, we compared the detected genes of single cells with that of bulk RNA. We found ~45.1 and ~62.6 % of the genes detected in bulk RNA were detected in a random single cell and 5 pooled cells, respectively (Methods, Fig. 2a, and Additional file 1: Figure S5). For the bulk RNA detected genes that were not detected in the random single cells, 70.3 % were low expression level genes (FPKM < 10) whereas only 27.6 % of the genes found in both bulk RNA and single cells have FPKM < 10. When we combined all 36 single cells together, the single cells covered 92.9 % of the genes detected in bulk RNA; 5322 genes were uniquely detected in the combined single-cell set and 1109 in the bulk RNA (Additional file 1: Figure S6). However 96.8 % of the 1109 bulk RNA unique genes were low abundance (FPKM < 10). The results reveal most of the bulk RNA uniquely detected genes were low abundance, so they were likely not detected in randomly single cells due to low or absent transcript in single cells. Notably, on average 9.3 uniquely detected genes per single-cell library (*N* = 36) had a strong signal (FPKM > 100), indicating some genes specifically expressed in rare populations can be detected only by single-cell RNA-seq. We evaluated gene detection sensitivity between MIRALCS and the tube-based method: 12,163 (*N* = 37, FPKM > 0) genes could be detected per cell by the MIRALCS, which was less than the tube-based method (14,050, *N* = 5, FPKM > 0, Fig. 2b), and comparable with that in downloaded single HeLa cells prepared by Fluidigm C1 system (6666, *N* = 220, FPKM > 0, Additional file 1: Table S13). To evaluate the gene detection efficiency for transcripts of different abundance, we examined the fraction of mRNA as a function of gene expression rank order and found they were consistent (*P* = 1, Student’s *t* test; Additional file 1: Figure S7).

In addition, we evaluated the influence of sequencing depth (from 0.1 to 8 million reads) on gene detection efficiency. The number of genes with FPKM > 15 did not vary with depth, and the number of genes with FPKM < 15 increased dramatically with increasing depth (Fig. 2c, Additional file 1: Figure S8). When the sequencing depth was above 1.5 million reads, the number of genes with FPKM > 1 remains nearly constant (Additional file 1: Figures S8, S9). Therefore we need not to consider the influence caused by sequencing depth in subsequent analysis since all libraries were sequenced more than 1.5 million reads.

To assess the accuracy of the MIRALCS, we added a known quantity of ERCC spike-in mRNAs. The estimated mean expression of these spike-ins was strongly correlated with input molecular number (*r* = 0.92, Fig. 2d), and the correlation coefficient increased to 0.96 when the spike-ins with an expected molecule number >1 per well were selected, indicating high accuracy of the MIRALCS. We modified the reaction conditions of the SMART-seq2 from 1–15 μl to 50 nl. To investigate any additional bias introduced by these modifications, we compared bias of strand, transcript coverage by position, transcript length and GC content for these two methods. To estimate the strand bias during PCR amplification, we compared the number of forward and reverse reads mapped onto the reference genome. The strong correlation between forward and reverse reads (mean *r* = 0.95, Additional file 1: Figure S10A) was comparable with bulk RNA (*r* = 0.97, Additional file 1: Figure S10B). We respectively estimated transcript coverage by position and fraction of detected genes in a range of transcript lengths, and found no differences from tube-based single cells and bulk RNA (*P* = 1, Student’s *t* test, Fig. 2e; *P* = 1, Student’s *t* test, Additional file 1: Figure S11A). To investigate GC bias, we determined the gene detection ratio over a range of GC content and observed no apparent bias (*P* = 1, Student’s *t* test, Additional file 1: Figure S11B). These results indicated that the MIRALCS was accurate in profiling single-cell transcriptomes.

To evaluate the reproducibility, we calculated the correlation coefficient of expression from external spike-ins and 10 pg RNA replicates. Firstly, we calculated the correlation coefficient between pairwise wells using the spike-ins expression and found the mean correlation coefficient was 0.95, revealing a high reproducibility of the MIRALCS platform (Fig. 2f, g, Additional file 1: Figure S12). Secondly, we also estimated correlation coefficients between pairwise 10 pg RNA replicates to assess the reproducibility, and observed that the gene expression consistency of the 5 replicated MIRALCS samples was much higher than that of the 3 repeated tube-based samples (*P* = 4.18 × 10^−6^, Student’s *t* test, Fig. 2h, i, Additional file 1: Figure S13). The better reproducibility of the MIRALCS could be due to more precise reagent loading.

### Single-cell RNA-seq reveals heterogeneity in HeLa S3 cells

The HeLa cell line is a valuable model for biological and molecular studies and we chose it for a pilot study of virus-infected tumors and cervical cancer research. Here, we described the transcriptome characteristics of HeLa S3 cells and investigated the heterogeneity in gene expression, alternative splicing, fusion and HPV-host transcript expression.

#### Differential mRNA abundance in HeLa S3 single cells

**Fig. 3:**
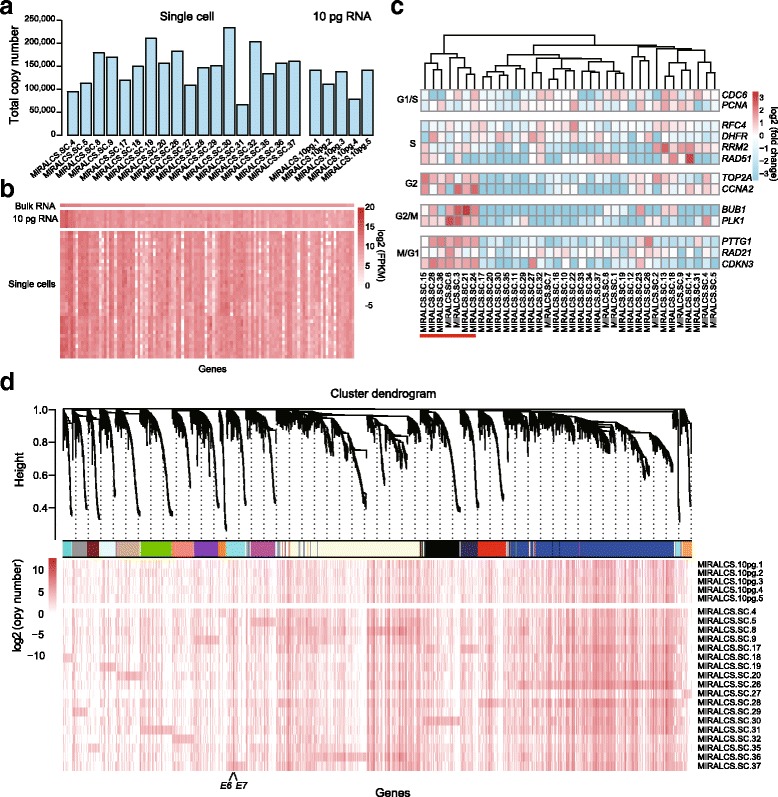
Heterogeneity of gene expression in HeLa S3 single cells. **a** The mRNA molecular number in single cells and 10 pg RNA replicates. **b** The heat map of the FPKM values of extremely highly expressed genes (FPKM > 500 in bulk RNA) in single cells and 10 pg replicates. **c** Single-cell subpopulations identification based on cell cycle relative genes. The cells with underline are in G2/M phase. **d** Gene co-expression modules derived from 19 single cells based on RNA molecular number (modules are distinguished by colors). The detailed of each module stands for were shown on Additional file 4: Table S7. The weighted gene correlation network was constructed using the WCGNA R package [38]

The normalized value of RPKM/FPKM and TPM are widely used in RNA-seq data analyses to indicate gene expression level. However, these values give a relative expression level rather than true transcript concentration, and can be affected by total RNA numbers in single cells [30]. To investigate the absolute mRNA molecular number of each gene, we used linear regression to calculate the relationship between FPKM and the actual added molecules according to the spike-ins [31] (Methods). We observed good agreement between the input number of spike-in RNA molecules and the corresponding FPKM values (Fig. 2d, Additional file 1: Figure S14). Using this normalization, we examined expression level distributions of all genes, and found the molecular number of most genes are from 1 to 60 in HeLa S3 cells, consistent with previous reports from lymphoblastic cells [31] (Additional file 1: Figure S15). We found striking cell-to-cell differences in the total transcript numbers of single cells (67,000–233,000), but relatively uniform numbers in the 10 pg RNA libraries (79,000–142,000) (Fig. 3a). We also found variable sizes of HeLa S3 cells (Additional file 1: Figure S16). According to previous reports [32, 33], variability of cell size contributes to the diversity of mRNA molecular number in cells. The average molecular number of mRNA in HeLa S3 cells was about double of that in a lymphoblastic cell line (~152,000 vs. ~80,000 [31]). To our knowledge, HeLa S3 cells are larger than lymphoblastic cells in size; thus, this phenomenon also supports the conclusion that cell size makes a contribution to the mRNA content of an individual cell.

#### Gene expression heterogeneity and co-expression network analysis of HeLa S3 single cells

We first selected high expression genes (FPKM > 100, Methods) to investigate gene expression heterogeneity. We found these highly abundant genes were enriched in pathways involved in metabolism of RNA and protein, and translation pathways in both bulk sample and single cells by using the Reactome analysis [34]. To investigate the gene expression heterogeneity in single HeLa S3 cells, we compared the gene expression profile at the single cell and population levels. We found that even for extremely high expressed genes of bulk RNA, they expressed a high range in single cells (FPKM > 500, Fig. 3b). To further analyze cell-to-cell gene expression variability, we examined the expression profile of 10 pg RNA replicates whose variation appears to be technical noise (Fig. 3b). Genes from 10 pg RNA samples display more stable expression than in single cells, indicating high heterogeneity in HeLa S3 cells. We further divided highly expressed genes into stably expressed (108 genes) and variably expressed (168 genes), based on the 10 pg dataset (Methods). However, we did not find any obvious difference in Reactome analysis result (Additional file 2: Table S5) and the ratio of housekeeping genes (40/168 vs. 27/108, *P* = 0.94, Chi-square test).

To further investigate the underlying factors/pathways triggering the heterogeneity of gene expression in HeLa S3 cells, we selected a higher variant gene set whose variance in single cells was > 6 folds of that in 10 pg RNA replicates. According to Reactome analysis, we found that the top three enriched pathway were cell cycle, immune system and cell cycle mitotic. This result supports the conclusion that cell cycle state makes a major contribution to heterogeneity of HeLa S3 cells, which also has been mentioned on recently published paper [35].

Therefore we next performed cell clustering to determine cell cycle phases based on single-cell gene expression. We clustered the single cells into groups based on the expression of phase-specific marker genes from a previous study [36]. In the clustering result, a group of 7 cells (19 %) displayed higher expression of G2/M phase marker genes (Fig. 3c). These 7 cells also showed a consistent pattern with our cluster result using a different set of cell cycle genes reported from another study [37] (Additional file 1: Figure S17). Flow cytometry resulted in a similar ratio of G2/M cells (17 %, Additional file 1: Figure S18). We performed differential gene expression analysis of the G2/M and non-G2/M groups, and identified 62 significant differentially expressed genes, including 1 lncRNA (*P* < 0.001, Additional file 3: Table S6).

To understand the co-expression relationships between genes at a systems level, we performed weighted gene co-expression network analysis (WGCNA) [38] using the molecules per cell estimated from above. We estimated variances within single cells and 10 pg RNA replicates, and selected genes whose variance in single cells was > 2-fold of that in 10 pg replicates. In total 4329 genes were selected for co-expression analysis by WGCNA, identifying 18 distinct co-expression modules and determined Reactome pathways for each module (Fig. 3d, FDR < 0.05, Additional file 4: Table S7). For the largest module (blue in Fig. 3d), genes were highly enriched in pathways of metabolism of RNA and protein and translation. Genes from the second largest module (light yellow in Fig. 3d) were enriched in cell cycle and immune system genes. Another interesting module including virus genes *E6*, *E7* will be discussed below.

#### Heterogeneity of splicing in HeLa S3 cells

**Fig. 4:**
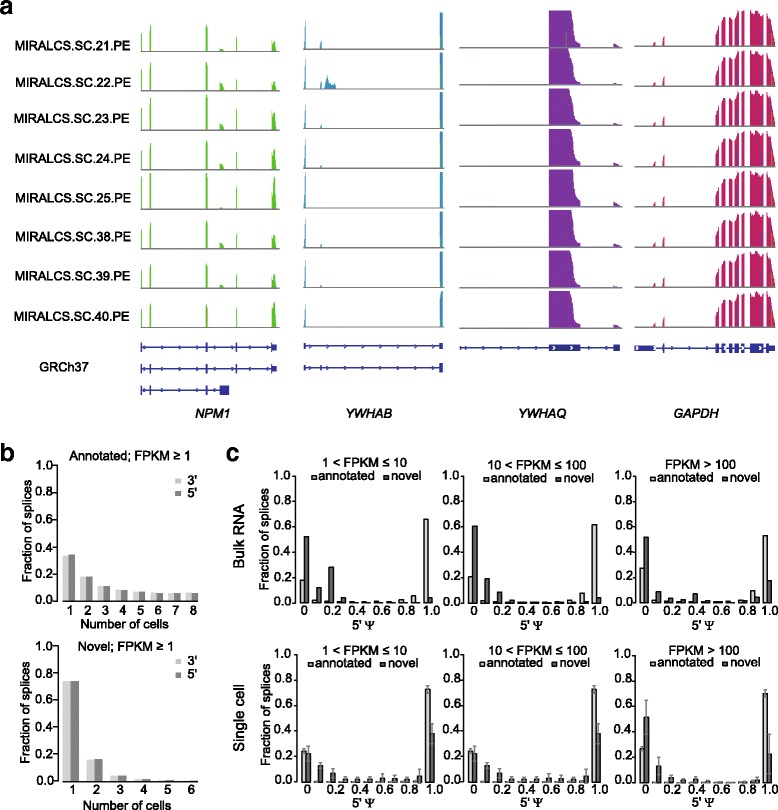
Heterogeneity of alternative splicing and distributions of splices in in single cells. **a** The sequencing depth for genes of *NPM1*, *YWHAB*, *YWHAQ* and *GAPDH* in single cells. **b** The frequency distribution of detected annotated and novel spliced junctions. **c** The distributions of the ψ scores of annotated and novel spliced junctions in the bulk RNA (*upper*) and single cells (*lower*)

Tumor specific alternative splicing isoforms have been reported in previous studies of cancer cells [39, 40]. So we investigated the alternative splicing of HeLa S3 cells both at the bulk level and single-cell level. To accurately detect alternative splicing events, we used paired-end sequencing data from eight single cells and one 5 ng total RNA. We divided splicing isoforms into known and novel isoforms according to the Ensembl database. We found that one third of genes expressed more than 1 isoform in both bulk and single cells, demonstrating that the majority of genes express only one isoform in HeLa S3 cells. We calculated the frequency of isoforms in single cells, and the number of isoforms with a frequency < 3 was much larger than those with a higher frequency (≥ 3) for both annotated and novel isoforms (Additional file 1: Figure S19A). This indicates that many splicing isoforms are only expressed in a small number of HeLa S3 cells. To further study splicing polymorphism in single cells [31, 41], we focused on highly expressed genes (mean FPKM > 100) to enhance detection accuracy, and found more than two thirds of genes expressed at least two isoforms in HeLa S3 cells. We focused on tyrosine kinase pathway related genes, which are the common targets for clinical drug treatment, and selected five genes to investigate in detail the alternative splicing heterogeneity in HeLa S3 cells. We found *ANXA2*, *NPM1*, *YWHAB*, and *YWHAZ* contained at least 2 different isoforms among different cells, while *YWHAQ* and the housekeeping gene *GAPDH* has only one isoform in all 8 cells (Fig. 4a). The variant forms in *YWHAB* and *YWHAZ* affect 5′ noncoding exons, while the alterations in *NPM1* and *ANXA2* affect coding exons at the C and N termini, respectively (Fig. 4a, Additional file 1: Figure S19B). Two isoforms of *YWHAB* were expressed in HeLa S3 cells, isoform 2 (NCBI) is expressed in all 8 single cells, while isoform 1 (NCBI) was only detected in 6/8 cells with a lower abundance. *NPM1* expressed 3 known isoforms with isoform 3 (NCBI) encoding the shortest protein and being expressed in some of the 8 single cells with variable abundance; isoform 1, the longest transcript, was highly expressed in all HeLa S3 cells, and isoform 2 was also expressed in all cells, but with an abundance of less than one tenth of that of isoform 1 (Fig. 4a).

To further quantify alternative splicing, we focused on paired donor-accepter splices with supported soft-clipped reads, and splices with at least one of the donor or acceptor sites annotated in GENCODE v19 (Methods). In addition to annotated splices, we detected considerably variability in the number of novel splices among single cells (72 to 780, Additional file 1: Figure S20). For both the annotated and novel splices, the majority were shared by a portion of the single cells (Fig. 4b). We used the intron-centric splice inclusion ψ score [42] to quantify the splices. The ψ score stands for the estimated expression ratio of the calculated intron-centric splice, in that case, ψ score equal to 1 means only one splice detected on this site. In bulk RNA, ψ scores of most novel splices were very low and most annotated splices were close to 1 (Fig. 4c). In single cells, ψ scores of annotated splices were similar to that in the bulk RNA. However, the fractions of ψ scores equal to 1 of novel splices were higher than those of bulk RNA among genes with FPKM less than 100. This was similar to that of bulk RNA with FPKM over 100, but single cells display a higher variance (Fig. 4c). This indicated that novel alternative splicing events tend to be more unique in single cells.

#### Heterogeneity of fusion transcript in HeLa S3 cells

RNA chimeric transcripts produced from fusion genes or two different genes by subsequent trans-splicing, and translated into chimeric proteins contribute to carcinogenesis [43]. Here, we used the transcriptome data to detect fusion transcript events in HeLa S3 cells at the bulk and single-cell levels. We detected 144 fusion transcript events in 8 single cells with different frequencies and only 1 event in bulk RNA (Additional file 1: Figure S21 and Additional file 5: Table S8). Of all the fusion candidates, we observed 33 intra- and 111 inter-chromosomal fusions.

The bulk sample detected fusion transcript *RPS6KB1-VMP1*, were also detected in 7/8 single-cell libraries and we validated this fusion event in bulk as well as in 8/8 additional single-cell cDNA (Additional file 5: Table S8), but it was negative in DNA. We also did not find any reads of the 4 × HeLa S3 whole genome sequencing data supporting this fusion event, suggesting that the *RPS6KB1-VMP1* fusion event was caused by a stable trans-splicing in HeLa S3 cells. This fusion also has been reported in breast cancer and several cancer cell lines including HeLa S3 [44, 45]; *RPS6KB*1 encodes the protein p70S6K that plays a key role in controlling the cell cycle, growth and survival [46]. Then we focused on the rest of the 143 fusion events uniquely found in single cells. *CEP89-PEPD* fusions were detected in 3/8 single-cell libraries, and validated both in bulk and 2/8 additional single-cell cDNA products, indicating a higher sensitivity for single-cell analysis heterogeneity of fusion transcript in HeLa S3 cells. Prolidase encoded by *PEPD* plays an important role in the recycling of proline for collagen synthesis and cell growth, the level of its activity in tissue and serum have been reported to be a marker of pancreatic cancer and associating with endometrial cancer and epithelial ovarian cancer [47–49].

### Diversity of HPV/human genome fusion in single HeLa S3 cells

**Fig. 5:**
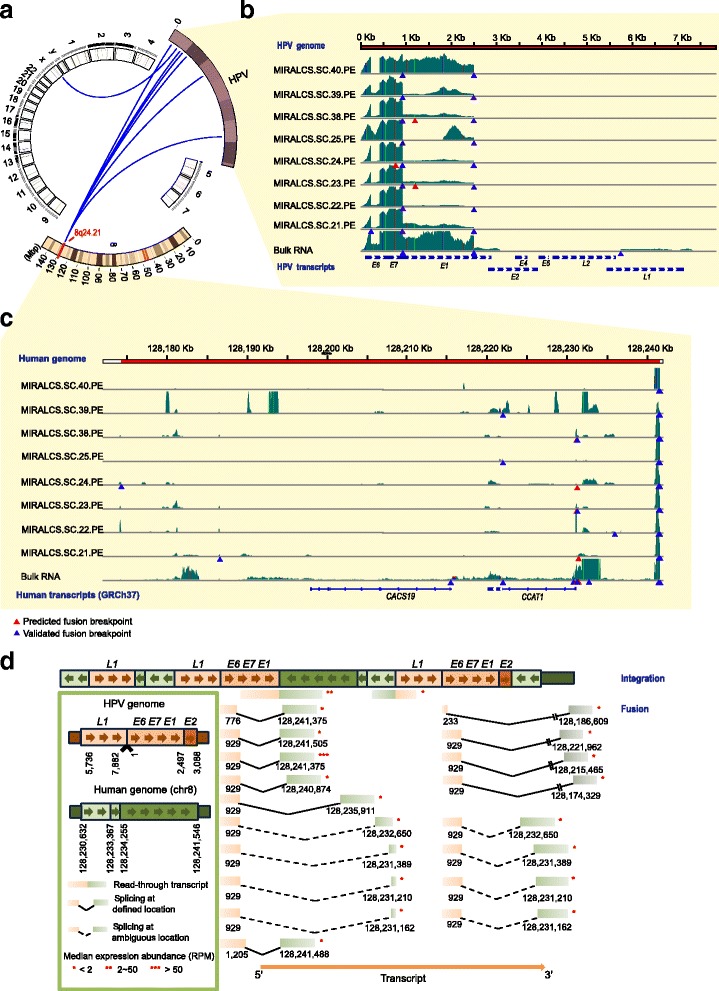
The landscape of the HPV-18/cellular fusion and diversity of HPV-host splicing and expression in HeLa S3 cells. **a** The overview of the HPV-18 cellular fusion based on HeLa cell transcriptome. *Blue lines* denote fusion events. **b** The read coverage of HPV-18 genome in single cells and the bulk RNA. *Colored vertical lines* denote nucleotides of SNPs detected in the transcriptome. *Light green*, A; *red*, T; *orange*, G; *blue*, C. **c** The read coverage of the host region on chromosome 8 in single cells and the bulk RNA. **d** The schematic diagram of the inferred HPV integration structure (*upper*) and splicing forms (*lower*). RPM stands for reads per million

Human papillomavirus (HPV) infection causes nearly all cervical cancer [12]. Previous studies have identified HPV-18 integration breakpoints in HeLa cell line using both DNA-seq and RNA-seq [26, 50, 51], but not at the single-cell level. We investigated the HPV/human breakpoints as “fusion” events using the paired-end data of eight single HeLa S3 cells and one 5 ng bulk RNA sample, and identified 16 distinct HPV-18/cellular fusion breakpoints (13 in single cells and 9 in the bulk RNA, Fig. 5a-c, Additional file 6: Table S9). The majority of these fusions were located at 8q24.21 which is a hotspot of HPV-18 integration [26, 50, 52] (Fig. 5a). A total of 10 and 6 events were located in intergenic regions and gene regions, respectively. Four sites were located at or close to the 5′ end of the gene *CCAT1*, which encodes a *MYC*-regulated long noncoding RNA (lncRNA) and effects cell cycle regulation and tumorigenesis [53, 54].

HPV-human fusion events called from RNA-seq are derived from HPV integration and splicing. To determine if these fusions are from integration or splicing, all 16 HPV fusions identified by RNA-seq were selected for validation on cDNA and DNA by PCR and Sanger sequencing, respectively. Eleven fusions were successfully validated in cDNA, while only 2 of them were also validated at the DNA level (Methods, Additional file 6: Table S9). And we identified the splice acceptor-donor sequences of 9/11 validated fusions and 3/5 of the remaining fusions (Additional file 1: Figure S22, gt-ag splicing); therefore, the majority of these fusions result from transcription initiation within HPV and splicing into flanking human DNA [55].

We also determined 4 HPV/human genome insertion sites in the HeLa S3 genome sequencing data consistent with earlier reports [26, 51] (Methods, Additional file 1: Table S10). To investigate the HPV integration and expression of fusion events at the single-cell level, we validated the 4 genome breakpoints and 5/11 RNA fusions in single-cell DNA and cDNA, respectively. We found all genome breakpoints were validated in all 10 additional single-cell DNA, while RNA fusions were validated in 64 additional single-cell cDNA samples with different frequencies (Additional file 6: Table S9), showing a diversity of splicing and expression of HPV-host transcripts in single cells. This data demonstrates that the integrations in the genome are invariant in all single cells, whereas the HPV-to-genome splicing events differed between individual cells.

By mapping the chromosome 8 HPV-genomic fusion transcripts onto the DNA sequence, we distinguished read-through and spliced transcripts resulting from the two independent HPV-18 genomic fragment copies, and we also detected multiple alternative splicing events (Fig. 5d). To further investigate the heterogeneity of HPV expression and splicing from these two integrated copies in HeLa S3 cells, we selected 5 “fusion” events and quantified the relative expression ratio of each transcript by using single RNA-seq data and qPCR results, at single-cell and bulk levels, respectively. In 64 single cells and one bulk cDNA, only 7/64 single cells and bulk cDNA expressed all 5 transcripts, the other single cells expressed 1 ~ 4 transcripts (Additional file 1: Figure S23A). We also found that for the majority of single cells, a read-through transcript (chr8: 128,241,546 − HPV: 2497) is the most abundant of the five transcripts (mean ratio 84.0 %). Another spliced transcript (chr8: 128,231,211 − HPV: 929) is expressed with a large range of transcript ratios (1 to 100 % in individual cells, Additional file 1: Figure S23). We observed the same proportional distribution pattern of these five transcripts from sequencing data and qPCR validation results (Additional file 1: Figure S23B), revealing that quantification from single-cell RNA-seq data is reliable. Therefore, we utilized the RNA-seq data to quantify all 15 fusions which were detected from the two integrated HPV copies (Fig. 5d). A considerable diversity and heterogeneity of expression of these transcripts were observed between single cells (Additional file 1: Figure S23C). We also found a spliced transcript (chr8: 128,241,375 − HPV: 929) showing much higher expression than others, and another read-through transcript (chr8: 128,241,546− HPV: 2497) expressed more stably. Therefore, we speculate that these two transcripts have a primary role in the HPV-18 tumorigenic process.

By mapping reads onto the HPV genome, we found two main splicing sites at nucleotide (nt) 233 and nt 929 in HPV, consistent with previous reported HPV isoforms [56]. We observed large differences in the ratio of splices at sites of nt 233 and nt 929 between cell populations (bulk RNA) and single cells (Additional file 7: Table S11). The majority of splices at site nt 233 were to nt 416 of HPV, while nearly all splices that occurred at site nt 929 were from the HPV-18 genome to the human genome (Additional file 7: Table S11). Splicing at nt 233 to nt 416 generates a truncated E6 protein (E6*I) which is thought to inhibit the function of complete E6 protein [57, 58]. It is interesting that only 1/8 cells predominantly express a complete E6, while all others express primarily the truncated E6. To our knowledge, this is first description of diversity and heterogeneity of HPV splicing and expression at the single cell level.

The HPV-18 E6 protein inactivates p53 [59] and E7 promotes the degradation of RB1 [60]. The expression of *E6* and *E7* is regulated by the E2 protein [61]. *E6* and *E7* were highly expressed in all cells (Fig. 5b), but we detected *L1*, *L2* gene expression only in the 5 ng bulk RNA library and 1/40 of the single-cell libraries. Interestingly, *E6* and *E7* were clustered into the same module in the gene co-expression analysis (Fig. 4d). Genes of this module were enriched in telomere maintenance and E2F mediated regulation of DNA replication, which plays important roles in HeLa oncogenesis [62, 63]. Genes in this module including *CDC25* [64], *PCNA* [65], *PLK4* [66], *BUB1B* [67] and *IRF1* [68] have been reported to be regulated by or interact with E6 and E7. We also performed gene classification in this module based on expression correlation to predict the genes influenced by E6 or E7, and found the *YWHAZ* gene, known to interact with *TP53,* tightly clustered with *E6* and *E7* (Additional file 1: Figure S24). So we speculate that additional genes within this module, including several lncRNAs (Additional file 8: Table S12), may be related to the viral infection/tumorigenesis process.

## Discussion

In this paper, we present a single-cell RNA preparation platform to realize high throughput, semi-automatic, full-length single-cell RNA preparation on a nanoliter-scale. Using this platform, we performed single-cell RNA-seq of a virally infected cell line and described a comprehensive understanding for the heterogeneity of HeLa S3 cells in gene expression, alternative splicing and fusion transcripts. We also provided cell classification based on cell cycle states and analyzed co-expression network modules of HeLa S3 cells. Furthermore, we characterized the diversity of HPV-18 expression and splicing in HeLa S3 cells at the single-cell level.

The new pipeline MIRALCS described here enables the preparation of full-length cDNA from more than 500 single cells per run in a microwell chip, which presents a higher throughput than that of the commercial instrument Fluidigm C1 (Additional file 1: Table S13). And MIRALCS generates comparable results with C1 for data quantity and gene detection number (MIRALCS vs. C1, 75 vs. 60 %, mean 7654 vs. mean 5619, FPKM > 1, Methods, Additional file 1: Table S13). Besides HeLa S3 cells, we have successfully used this platform to prepare single-cell RNA of biopsy material from a variety of samples such as bladder cancer tissue, liver cancer tissue and B cells, with a higher success rate compared with a tube-based approach (data not shown). To approach absolute quantitation of mRNA copy number we used a spiked-in RNA. However we note that this method has limitations due to loading of consistent spike-in amounts and efficiency of amplification of individual genes. In addition, the MIRALCS method could be further improved. The current version of MIRALCS only automates the amplification of single-cell cDNA, while the library construction is finished in single tubes, so we are working on performing library construction in the same well as the cDNA amplification, to further improve the automation of single-cell RNA preparation.

In this paper, we investigated virally infected cells by single-cell RNA-seq. By the analysis of HPV-18 transcripts, we observed the diversity of HPV-18 splicing of sequences integrated in the host genome. Similar studies could also be carried out to study clinical cervical cancer as well as other virally induced cancers. Investigating of viral gene expression could be used to monitor infection and progression of virus induced cancers [50, 69].

Single-cell transcriptome analysis can also be used to identify co-expressed genes [31]. In our data we identified a cluster of co-expressed genes containing both the *E6* and *E7* viral oncogenes, along with 281 cellular genes. In this cluster, apart from some known genes regulated by or interacted with E6 and E7, the additional cellular genes including lncRNAs are candidate genes potentially interacting with E6 and E7 and contributing to viral transformation. Of course, further functional experiments are needed to validate these genes.

In summary, MIRALCS is an improved method for single-cell transcriptome analysis. Using this platform, we realized a transcriptome study in HeLa S3 cell at the single-cell level, and presented the heterogeneity of gene expression, alternative splicing, fusion transcript and HPV-host splicing in this virus-infected cell line. Our data provides further understanding of this widely used biological and molecular model as well as a pilot study of single-cell RNA-seq in virally infected cancers.

## Methods

### Cell culture, single-cell suspension preparation and RNA extraction

HeLa S3 cell line was purchased from American Type Culture Collection (ATCC, CCL-2.2) and stored at −80 °C. After anabiosis, the cells were cultured in DMEM medium (GIBICO) supplemented with 20 % (*v/v*) FBS (GIBICO), 1 % L-glucose and 1 % nonessential amino acid, at 37 °C in a humidified incubator containing 5 % (*v/v*) CO_2_. Cells were collected into a 1.5 ml tube and the concentration of cells was adjusted to 2 ~ 8 cells/μl in 20 % Percoll solution. Percoll solution was purchased from Pharmacia, and mixed with 10 × Phosphate Buffer Solution (PBS) with ratio 9:1 to generate 100 % Percoll solution. And the 20 % Percoll solution was prepared by 1 × PBS and 100 % Percoll solution mixed with ratio 8:2. Total RNAs from HeLa S3 cell populations were extracted by an RNeasy plus mini kit (Qiagen) according to the manufacturer’s instructions.

### cDNA synthesis and amplification

The cDNA preparation of regular tube-based method for HeLa S3 single cells, 10 pg total RNA and 5 ng total RNA completely followed the SMART-seq2 protocol [28]. The amplified cDNA of HeLa S3 single cells and total RNA (1, 10, 40 and 160 pg) prepared by MIRALCS followed a modified SMART-seq2 protocol with the following steps. For some single cells and all 10 pg total RNA replicates, External RNA Controls Consortium (ERCC) spike-in mRNAs (Ambion, Life Technologies) were added into lysis buffer (mean 12,463 or 2493 copies per well). Samples and all reagents were dispensed into a customized 200 nl microwell chip (WaferGen Biosystems) by multiple sample nanoliter dispensers (MSND, WaferGen Biosystems). Firstly, 50 nl lysis buffer (10 % Triton X-100 0.5 nl, 40 U/μl RNase Inhibitor 1.25 nl, 10 μM Oligo-dT Primer 12.5 nl, 10 mM dNTP Mix 12.5 nl and spike-in RNAs or nuclease-free water 23.25 nl) was dispensed into every microwell on the chip, then 50 nl samples of HeLa S3 cells with concentration of 8 cells/μl, or negative control (20 % Percoll solution) or total RNA positive controls (1, 10, 40 and 160 pg/50 nl) were added into the wells. After cell lysis (72 °C for 3 min and 4 °C for 5 min), reverse transcription mixed solution (200 U/μl Super Script II Reverse Transcriptase 6 nl, 5× SuperScript II First-Strand Buffer 16 nl, 5 M Betaine 16 nl, 100 mM MgCl_2_ 7.2 nl, 100 μM template-switching oligos 0.8 nl, 100 mM DTT 2 nl and 40 U/μl RNase inhibitor 2 nl per well) was dispensed into wells, then the reverse transcription reactions were carried out (42 °C for 90 min, 2 cycles of 50 °C for 2 min and 42 °C for 2 min, and then incubated at 70 °C for 15 min, 12 °C for 5 min) on thermal cycling instrument (Prime). At last we added PCR reaction buffer (2× KAPA HiFi HotStart ReadyMix 41.67 nl, 10 μM IS PCR Primer 0.83 nl, Nuclease-free water 5 nl and 20× SYBR Green I 2.5 nl per well) into wells by MSND, and amplified cDNA on SmartChip™ Real-Time PCR Cycler (WaferGen Biosystems). During cDNA amplification, SmartChip™ Real-Time PCR Cycler monitored the fluorescence of SYBR Green I and outputted the curves of fluorescence, the values of cycle threshold (Ct) and melting temperature (Tm).

In the process of sample and reagent dispensing, the reagents firstly was added into 36 wells of 384–well plate following the MSND operation manual, and then the reagents in each well of 384-well plate were transported into 144 wells of 5184-well microwell chip by MSND. In this article, 5/36 wells were added as negative control and positive controls, so only 31 wells containing HeLa S3 cells were dispensed into 4464/5184 wells of microwell chip. More detailed operation steps of this platform can be found in Additional file 1: Note 1.

### Cell distribution calculation and target wells confirmation

To observe the cell distribution on microwell chip at different cell concentrations, we dispensed the cell suspension (cell stained by SYBR Green I) on a diaphanous plastic film instead of the chip by MSND, which enabled us to calculate the cell distribution under the microscope (Additional file 1: Figure S25).

Target wells containing cell cDNA products were confirmed by Agilent 2100 Bioanalyzer. The 2100 result of a real target well showed a main fragment from 500 to 3000 bp with a peak at 1 ~ 2 kb, similar to the RNA positive control; and the 2100 result of a real non-target well showed no fragments or only fragments shorter than 200 bp, which were primer dimers, similar to the negative control (Additional file 1: Figure S3).

### Library construction and sequencing

For the tube-based method, amplified cDNA products were purified by 1 × Agencourt AMPure XP beads (Beckman Coulter). A total of 2 ng purified cDNA products from each sample were used as the starting amount for library preparation. For the MIRALCS method, amplified cDNA was extracted by an automatic extractor from the chip to 96-well plate and diluted from 200 nl to 5 μl. And 3 μl cDNA products without purification were directly used for library construction. The libraries were prepared by TruePrep™ Mini DNA Sample Prep Kit (Vazyme Biotech) according to the instruction manual and each sample was labelled with a barcode. All of the samples (40 single cells and five 10 pg total RNA replicates prepared by MIRALCS; and five single cells, three replicates 10 pg total RNA and one 5 ng bulk RNA from populations of HeLa S3 cells prepared by tube-based SMART-seq2 approach) were sequenced on Illumina HiSeq 2000 sequencing system. Paired-end and single-end sequencing strategies were both used for different analysis purposes (Additional file 1: Figure S1, Tables S3, S4).

### Public data set access

Human (Homo sapiens) reference genome sequence (Hg19, GRCh37, Feb, 2009) was downloaded from University of California Santa Cruz Genome Bioinformatics [70], and the information of chrY was removed before the analysis. The transcriptome reference annotation GTF file (Ensembl GRCh37.75) was downloaded from the Ensembl database [71]. The GENCODE annotation file (v19) was downloaded from the GENCODE project [72]. The HPV-18 reference genome sequence (GenBank: NC_001357.1) was downloaded from the National Center for Biotechnology Information [73].

### Processing the mRNA sequencing data

The reads with the adaptor or poly-A sequences were filtered out from the raw FASTQ data before alignment using in-home C++ scripts. Besides, the low quality reads which the N rate > 0.01 and the low quality base (quality < 5) rate > 0.5 were also filtered out. Given the different alignment efficiencies of software, clean reads were aligned using TopHat2 [74] (v2.0.12) with Bowtie [75] (v0.12.9.0) for single-end reads (49 bp) and Bowtie2 [76] (v2.1.0.0) for paired-end reads (90 and 150 bp). The indexes of Bowtie and Bowtie2 were built using the combination of the human genome, the HPV-18 genome and ERCC spike-in mRNAs’ sequences. The parameters for Bowtie were -g 1 -N 1 --solexa1.3-quals --segment-length 24 --segment-mismatches 1, and the parameters for Bowtie2 were -g 1 --read-gap-length 3 --read-edit-dist 3 --b2-very-sensitive --solexa1.3-quals --segment-length 30 --segment-mismatches 1. Gene expression levels were quantified as fragments per kilobase of gene per million mapped reads (FPKM). Read counts were calculated by feature-count (Rsubread [77], v1.16.1), and FPKM values were calculated using edgeR [78] with the reference annotation GTF file. The public HeLa single-cell RNA-seq data generated by Fluidigm C1 platform were downloaded from NCBI (Accession: PRJDB3416). The same pipeline of reads filtering, alignment and FPKM calculation were performed on these single cells. The single cells with mapped reads < 1.5 million were filtered out.

### Evaluation for the performance of the MIRALCS system

To evaluate the sensitivity and efficiency of the system, BAM files from high-coverage sequencing data (from 8 single cells with more than 10 million reads and 5 ng bulk RNA) were downsampled by randomly selected reads at 17 sequencing depths (0.1 million reads to 0.9 million reads; 1 million reads to 8 million reads) using a Perl script, and downsampled results were then re-processed. The downsampled 2 million reads files of single cells were randomly selected and processed for gene detection in single cells and bulk RNA, and one single-cell library with less than 2 million reads was discarded from this evaluation analysis. In addition, the detected genes from the merged datasets from 5 randomly selected single cells (downsampled 0.4 million reads per cell) were also compared with those from bulk RNA (downsampled 2 million reads) and were repeated for 5 times (Fig. 2a and Additional file 1: Figure S5). To evaluate the coverage bias, the genes with only one isoform were selected, and divided into 100 windows from 5′ end to 3′ end to calculate the fraction of the depth. One-side Student’s *t* test was used for the comparison between the correlation coefficients of tube-based and MIRALCS 10 pg replicates. Two-side student’s test was used for other p values calculation on this paper unless additional mentioning. Pearson correlation coefficient was calculated for all the correlation analysis.

For each sample with spike-ins, the linear regression forcing the regression through 0 (to avoid the assignment of positive copies per cell of genes with 0 FPKM values) was used to calculate the relationship between log2 transformed FPKM values and log2 transformed actual added copies of the spike-ins. Only spike-ins with molecules number more than 5 and FPKM values more than 0 were used. The FPKM values of all genes were converted to approximate copies using the linear regression method on a log2 scale.

### Alternative splicing and fusion detection

Considering the accuracy of alternative splicing detection, only the paired-end sequencing data (8 single cells and the 5 ng RNA) with PCR replicates removed using samtools (14) were used. The 5′ and 3′ splicing inclusion ψ scores [42] were calculated using the IPSA package [79] as followed:

$$ \begin{array}{c}\hfill {\psi}_5\left(\mathrm{D},\mathrm{A}\right)=\frac{N_{reads}\left(D,A\right)}{\sum_{A_i\in A}{N}_{reads}\left(D,{A}_i\right)}\hfill \\ {}\hfill {\psi}_3\left(\mathrm{D},\mathrm{A}\right)=\frac{N_{reads}\left(D,A\right)}{\sum_{D_i\in D}{N}_{reads}\left({D}_i,A\right)}\hfill \end{array} $$

Where D and A refer to the donor and acceptor splice sites respectively. The *N*_*reads*_ refers to the number of the reads crossing the donor or acceptor sites after removing the PCR duplication. To remove the potential artifacts during the experimental and sequencing procedure, only the known splice junctions and novel junctions that contained at least one site annotated in GENCODE v19 were retained for further analysis. The paired splice junctions for which neither the donor nor acceptor exists in GENCODE v19 were discarded.

To analyze the different isoforms, Cufflinks [74] (v2.1.1) was used with the parameter –u and Cuffcompare to obtain the known transcripts set and the novel transcripts set which were not found in the annotation file. The reads distribution of the genes in *ANXA2*, *NPM1*, *YWHAB*, *YWHAQ*, *YWHAZ* and *GAPDH* was visualized using the Integrated Genome Viewer [80].

To ensure the accuracy of fusion detection, the same data set in splicing calling was used. TopHat-fusion [81] (v2.0.12) was used with parameters --fusion-search --fusion-min-dist 100,000. TopHat-fusion-post was then used with the parameters --num-fusion-reads 1 --num-fusion-pairs 0 --num-fusion-both 5. The breakpoints within less than 10 bp were merged considering the mismatches around the breakpoints.

### HPV/cellular fusion detection

For the HPV genome was included in Bowtie/Bowtie2 index, the calculations of HPV genes expression and the detection of HPV/cellular fusion breakpoints was performed during the processing of the sequencing data. The fusions were filtered as follows: 1) at least 5 mate pairs that had one end spanning the fusion; 2) at least 10 spanning reads; 3) at least 1 spanning read that covered the sequence length of each side of the breakpoint for more than 65 bp. The fusion breakpoints within 10 bp were then merged. Previous research [26] has reported the haplotype of HeLa S3 cell line and the HPV integration. Considering the differences in the same cell lines from different labs, the sequencing data of the mixed gDNA from our HeLa S3 cell line with ~4× sequencing depth was aligned to the reported haplotype (data not shown). The haplotype was modified according to our sequencing data. Therefore, the HPV/cellular fusions were compared to the modified haplotype, and a fraction of fusions were consistent. For the fusions that did not exist in the haplotype, splicing donor-accepter signal GT-AG was found at the fusion boundaries (Additional file 1: Figure S23).

### Heterogeneity analysis of single cells

Genes with FPKM more than 500 in bulk RNA were selected for the mosaic expression analysis. To investigate the different functions of genes with high variation and low variation of expression, the ratio of (variance of MIRALCS-SC)/(variance of MIRALCS-10 pg) of 442 genes with FPKM more than 200 in bulk were calculated. Among these genes, 168 genes with the ratio more than 6 were considered as highly differently expressed genes, and 108 genes with the ratio less than 2 were treated as stably expressed genes. The Reactome [34] enrichment analysis was performed using the MSigDB [82].

For the two different cell cycle phase-specific genes sets [36, 37], genes with the mean FPKM value of single cells more than 10 were retained to avoid false positives caused by low mean FPKM values. Using these genes, single cells were classified into different clusters based on the hierarchical clustering method.

### Co-expression analysis

The genes expressed at more than one estimated copy per cell in at least one cell were retained. To minimize the influence of the stochastic differences during the experimental and sequencing procedures, genes with a variance of copies less than twice of that of the 10 pg replicates were filtered out. The co-expression networks was constructed from the single cells using the WGCNAR package with β = 8 and a minimum module size of 25 genes. The Reactome enrichment analysis was performed using the MsigDB.

### Validation of detected fusions

The selected fusion breakpoints (human-human and HPV-human) were validated by PCR in bulk HeLa S3 cDNA, DNA and single-cell cDNA and DNA. We designed the PCR primers on the basis of the paired-end assembled fragments, in which one primer was located in the left gene of fusion and the other in the right gene of the fusion. Sanger sequencing was then used for the PCR validated products on an Applied Biosystems 3730 DNA analyzer (Life Technologies, Inc.). For fusion transcripts frequencies validation (*RPS6KB1-VMP1* and *CEP89-PEPD*), 8 single cell cDNA were used for PCR validation. For HPV-host fusions, qPCR was used in additional 64 single-cell products to further validate the breakpoints frequency (Additional file 1: Figure S1).

## Availability of supporting data

The raw sequencing data in the fastq format is available in the database of Genotypes and Phenotypes (dbGaP) as an approved sub study of the HeLa Cell Genome sequencing Studies, phs000640, and the gene expression data from this study hosted in the GigaScience Repository, GigaDB [83].

## Additional files

Additional file 1: **Supplementary information.** (PDF 2730 kb)
Additional file 2: Table S5. The Reactome results of variably expressed genes and stably expressed genes (FDR<0.05). (XLSX 21 kb)
Additional file 3: Table S6. Differential expressed genes between the G2(M) and non-G2(M) group of cells. (XLSX 11 kb)
Additional file 4: Table S7. The Reactome results of defined co-expression modules. (XLSX 40 kb)
Additional file 5: Table S8. Detailed information on fusion sites detected from RNA data. (XLS 143 kb)
Additional file 6: Table S9. Detailed information of HPV-human fusion sites detected from RNA data. (XLS 35 kb)
Additional file 7: Table S11. The reads number and ratio of different types of 233 bp and 929 bp in HPV genome. (XLS 11 kb)
Additional file 8: Table S12. Genes clustered with the *E6* and *E7* in co-expression analysis. (XLSX 12 kb)

## Abbreviations

MIRALCS: Microwell full-length mRNA amplification and library construction system
MSND: Multiple sample nanoliter dispensers
WGCNA: Weighted gene co-expression network analysis
