# Supplementary material for: Full-length single-cell RNA-seq applied to a viral human cancer: applications to HPV expression and splicing analysis in HeLa S3 cells
Source: Gigascience. 2015 Nov 5;4:51. doi: 10.1186/s13742-015-0091-4 (PMC4635585; doi:10.1186/s13742-015-0091-4)
Supplement: Additional file 1: — Supplementary information. (PDF 2730 kb) [file 13742_2015_91_MOESM1_ESM.pdf]

Full-length single-cell RNA-seq applied to a viral human cancer: Applications to HPV expression and splicing analysis in HeLa S3 cells

Liang Wu[†1], Xiaolong Zhang[†1,2], Zhikun Zhao[†1,3,4], Ling Wang[†5], Bo Li[1], Weijian Rao[1], Guibo Li[1], Michael Dean[6], Yanhui Wang[1], Qichao Yu[7], Xinxin Lin[1], Zhanlong Mei[1], Yang Li[1], Runze Jiang[1], Huan Yang[1], Fuqiang Li[1], Guoyun Xie[1], Liqin Xu[1], Kui Wu[1], Jie Zhang[1], Jing Wei[1], Jianghao Chen[5], Ting Wang[5], Karsten Kristiansen[8], Xiuqing Zhang[9], Yingrui Li[1,10], Huanming Yang[1,11], Jian Wang[1,11], Yong Hou[*1,8], Xun Xu[*1]

Supplementary Information

**Supplementary Figure 1**	The flowchart of sample preparation and analysis
**Supplementary Figure 2**	The distribution of cells in wells for different cell concentrations
**Supplementary Figure 3**	The melting temperature (Tm) and cycle threshold (Ct) of different samples including 1 pg, 10 pg, 40 pg, 160 pg RNA after SYBR Green I based cDNA amplification
**Supplementary Figure 4**	Typical Agilent 2100 bioanalyzer detection results of the products identified by Tm and Ct values extracted from micro-wells
**Supplementary Figure 5**	The overlap of genes detected in each single cells and in bulk RNA
**Supplementary Figure 6**	The overlap of genes detected in combined single cells and bulk RNA
**Supplementary Figure 7**	The fraction of genes detected at different expression levels
**Supplementary Figure 8**	Gene detected numbers along expression level with different sequencing depths.
**Supplementary Figure 9**	Saturation curves of gene detection number by different sequencing depths (FPKM > 1)
**Supplementary Figure 10**	Strand bias of the RNA libraries
**Supplementary Figure 11**	The gene distribution by different length of transcripts and GC content
**Supplementary Figure 12**	The correlation coefficients of pair-wise ERCC spike-ins in different wells
**Supplementary Figure 13**	The correlation coefficient of pair-wise 10 pg RNA replicates
**Supplementary Figure 14**	The linear regression relationship between FPKM and real mRNA copy number of spike-ins of one randomly selected single cell
**Supplementary Figure 15**	Distribution of gene expression levels in estimated copies per cell in single cells and 10 pg RNA replicates
**Supplementary Figure 16**	The variable sizes of HeLa S3 cells observed by microscope
**Supplementary Figure 17**	Cell clustering according to phase-specific expressed genes.
**Supplementary Figure 18**	Cell cycle analysis by flow cytometry
**Supplementary Figure 19**	Alternative splicing isoform distribution in single cells and reads

	mapping of gene *AXAN2* and *YWHAZ*
**Supplementary Figure 20**	The number of detected novel splices in single cells
**Supplementary Figure 21**	The frequency of detected gene fusions in single cells
**Supplementary Figure 22**	Splicing donor-accepter signals detected at the HPV-18/cellular fusion breakpoints
**Supplementary Figure 23**	Quantification of HPV/human fusions
**Supplementary Figure 24**	Clustering of correlation coefficients of genes in the module which contains *E6* and *E7*.
**Supplementary Figure 25**	Cells dispensed on a plastic film under the microscope
**Supplementary Table 1**	Information of the sample-preparation platform and object (single cell or total RNA) of each library used
**Supplementary Table 2**	Information of RNA-seq data
**Supplementary Table 3**	Cell settling rate in Percoll solution of different concentrations
**Supplementary Table 4**	The Ct and Tm values of samples used to validate the selection mechanism
**Supplementary Table 5**	The Reactome results of variably expressed genes and stably expressed genes
**Supplementary Table 6**	Differential expressed genes between the G2(M) and non-G2(M) group of cells
**Supplementary Table 7**	The Reactome results of defined co-expression modules
**Supplementary Table 8**	Detailed information on fusion sites detected from RNA data
**Supplementary Table 9**	Detailed information of HPV-human fusion sites detected from RNA data
**Supplementary Table 10**	Reads support by HeLa S3 cell line genome sequencing
**Supplementary Table 11**	The reads number and ratio of different types of 233bp and 929bp in HPV genome
**Supplementary Table 12**	Genes clustered with the *E6* and *E7* in co-expression analysis
**Supplementary Table 13**	The comparison between MIRALCS and Fluidigm C1 system.
**Supplementary Table 14**	The components of lysis buffer
**Supplementary Table 15**	The components of reverse transcription reaction buffer
**Supplementary Table 16**	The components of cDNA amplification reaction buffer
**Supplementary Note 1**	Operation steps of MIRALCS

Supplementary information

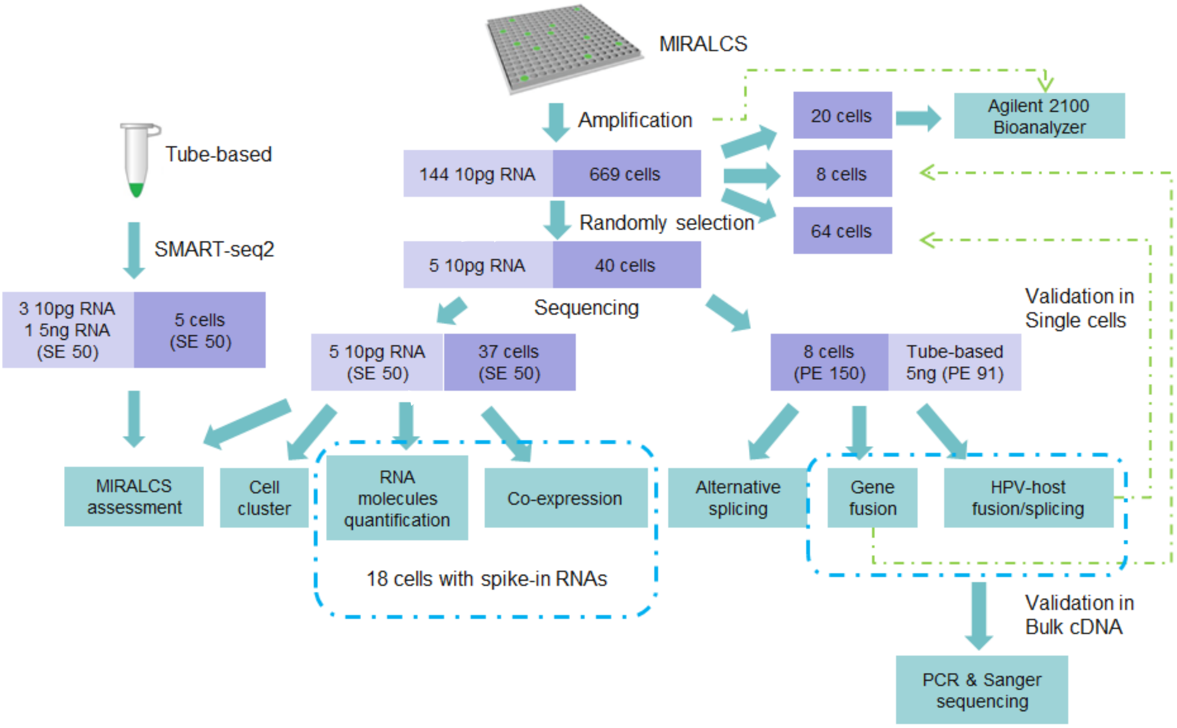

Supplementary Figure 1. The flowchart of sample preparation and analysis.

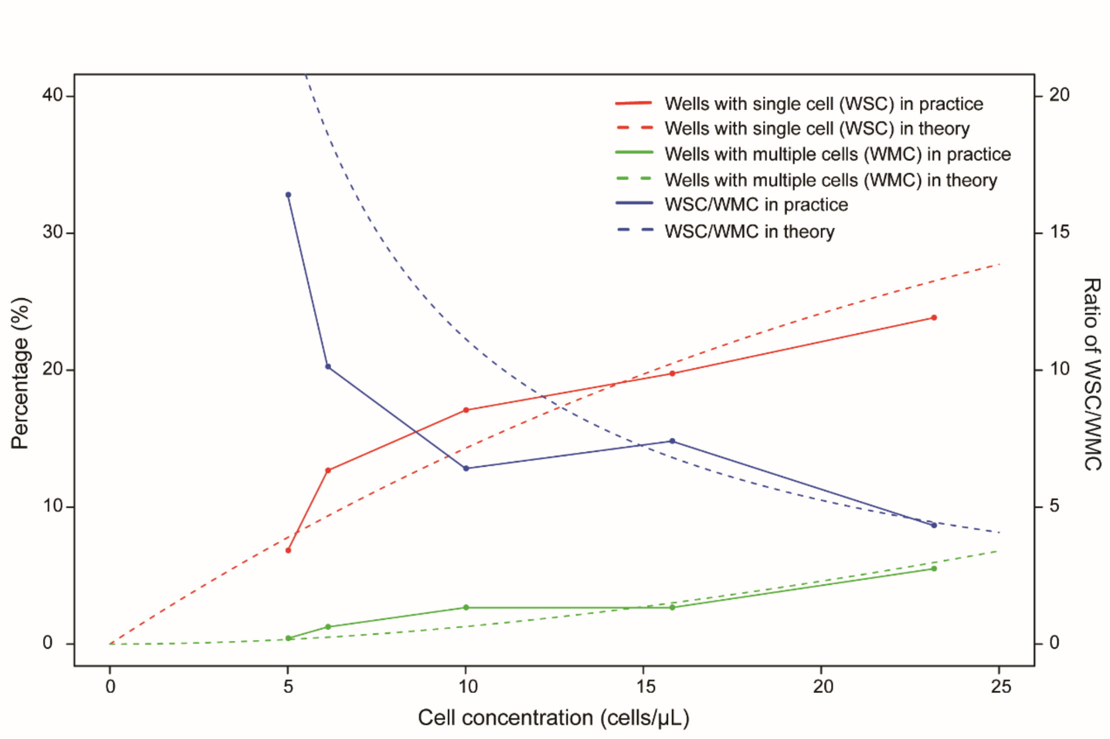

Supplementary Figure 2. The distribution of cells in wells for different cell concentrations. Red lines indicate the percentage of wells with single cells; green lines the percentage of wells with multiple cells; blue lines represent the ratio of wells with single cells to that of multiple cells. The solid line is the experiment result, while the dotted line is the theoretical result. The theoretical result follows the Poisson equation with modification of λ to ~$\lambda/2.951$.

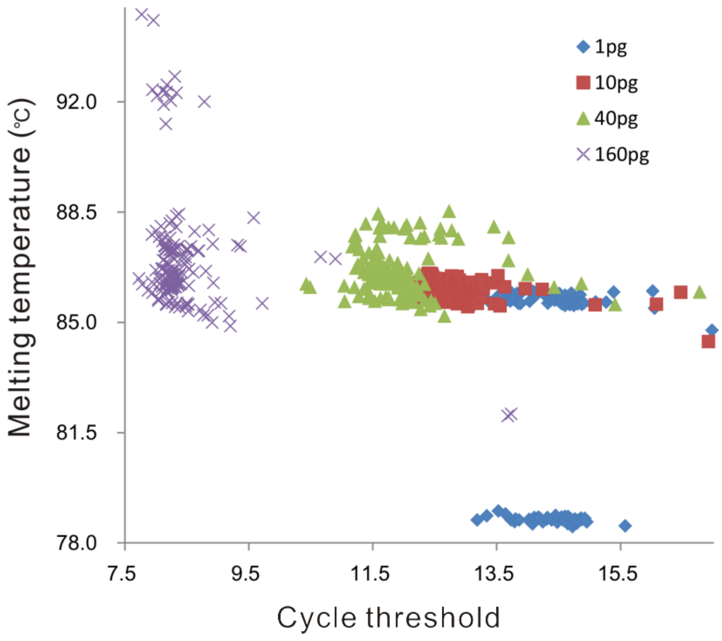

Supplementary Figure 3. The melting temperature (Tm) and cycle threshold (Ct) of different samples including 1 pg, 10 pg, 40 pg and 160 pg RNA after SYBR Green I based cDNA amplification.

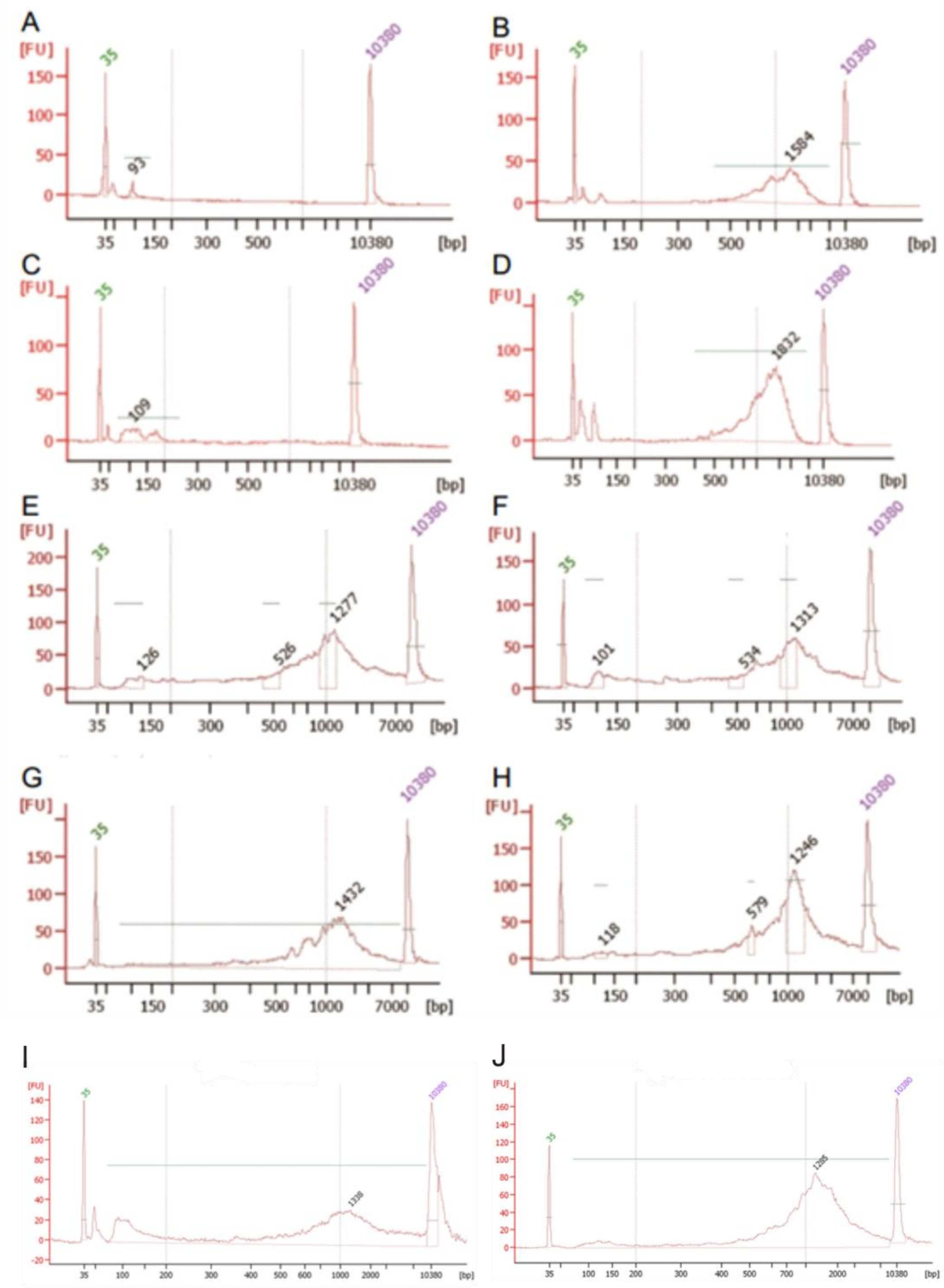

Supplementary Figure 4. Typical Agilent 2100 bioanalyzer detection results of the products identified by Tm and Ct values extracted from micro-wells. (A) Negative control of 20% Percoll solution without cells; (B) Positive control of total RNA extracted from HeLa S3 cells with a concentration of ~10 pg/well; (C) Expected non-target well without HeLa S3 cell; (D) Expected target well with HeLa S3 cell; (E) Expected target well of a fresh urinary bladder cancer tissue cells; (F) Expected target well of fresh human bladder cells; (G) Expected target well with a CLL cell identified; (H) Expected target well of a frozen mouse urinary cell identified; (I) Expected target well of frozen human bladder cell; (J) Expected target well of frozen mouse liver cell.

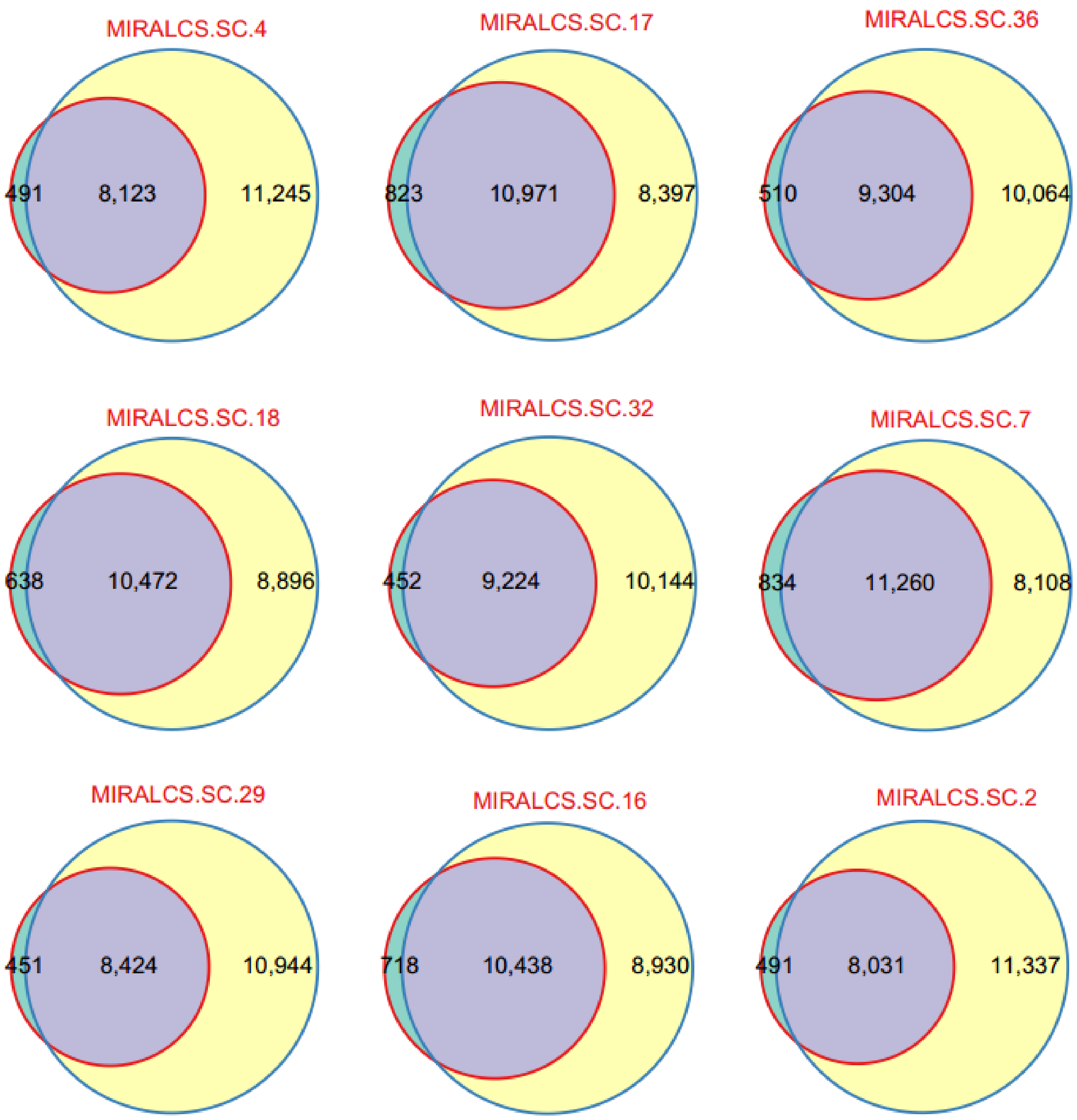

Supplementary Figure 5. The overlap of genes detected in each single cells and in bulk RNA. In total 9/37 randomly selected Venn diagram comparisons are shown. The smaller circles represent single cells, and the larger ones represent bulk RNA.

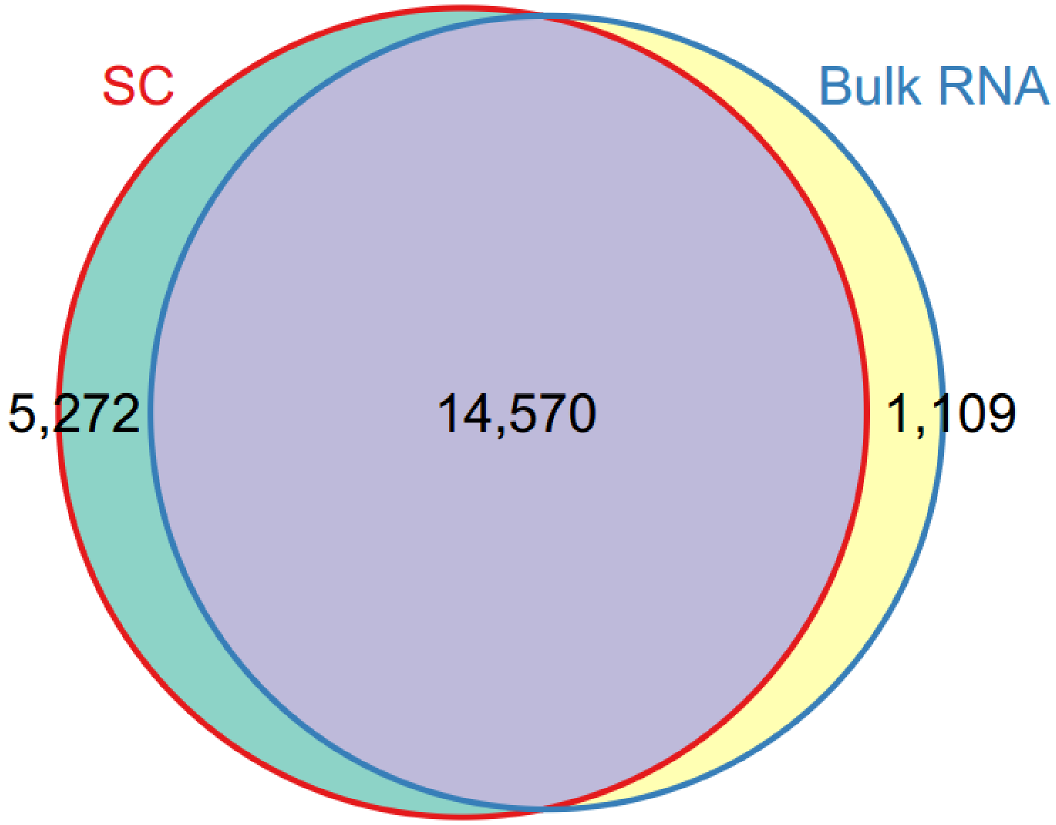

Supplementary Figure 6. The overlap of genes detected in combined single cells and bulk RNA. The circle with the red line represents the genes detected in any of 37 single cells, and the blue one represents genes detected in bulk RNA.

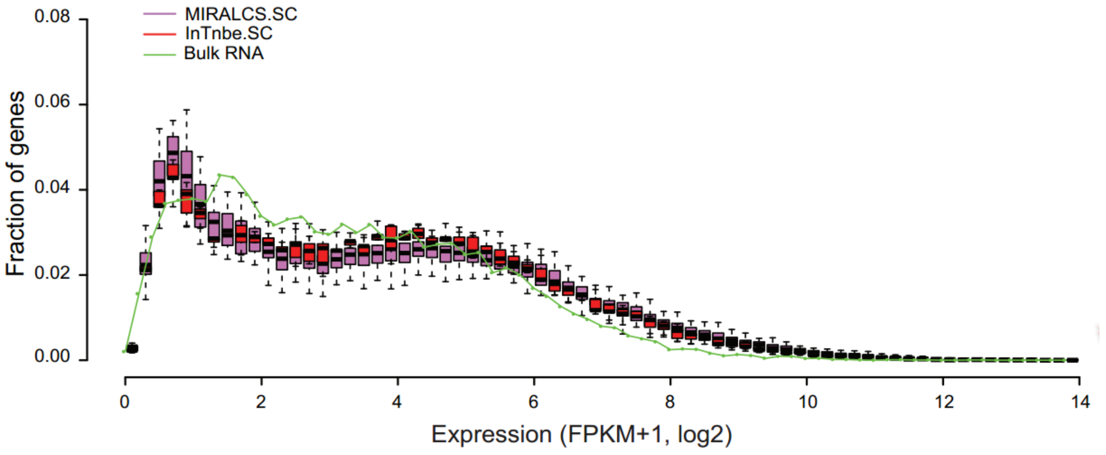

Supplementary Figure 7. The fraction of genes detected at different expression levels.

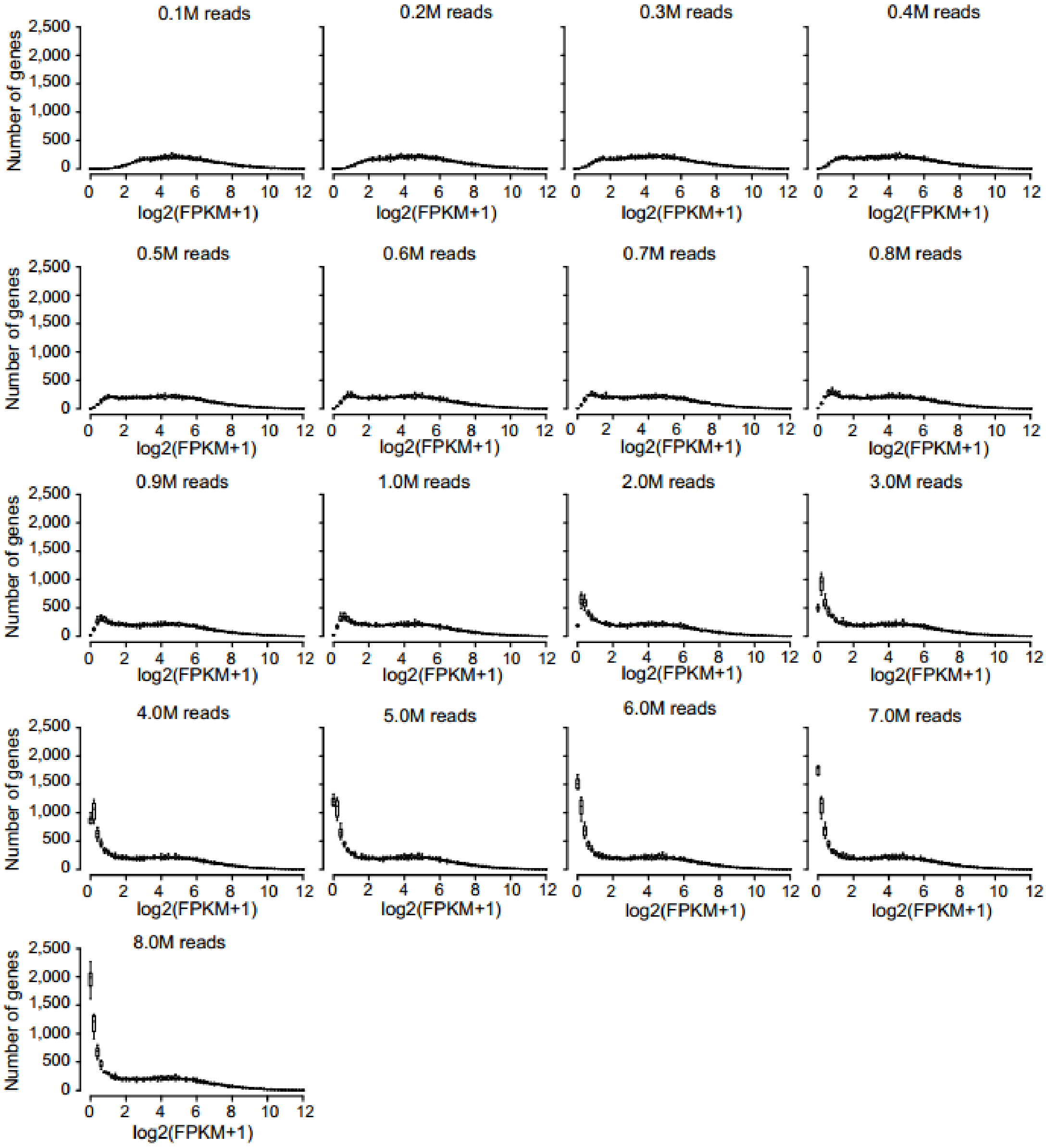

Supplementary Figure 8. Gene detected numbers along expression level with different sequencing depths. Each figure was generated from randomly downsampled unique mapping reads from the same group of single cell libraries.

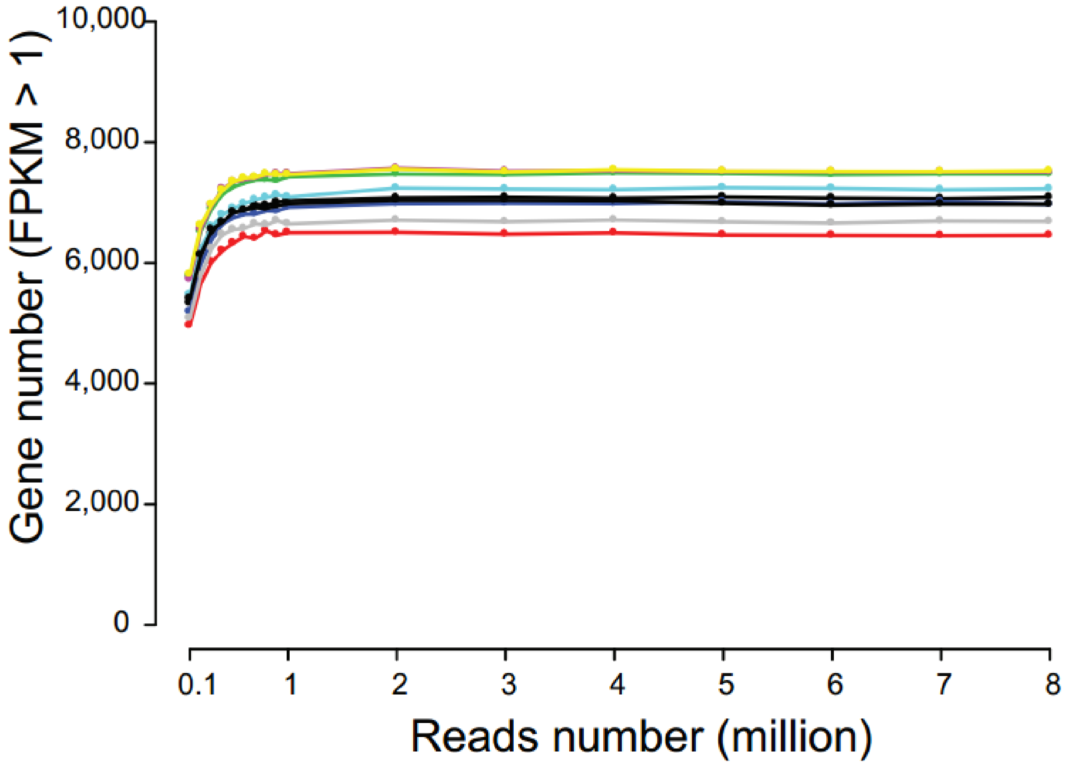

Supplementary Figure 9. Saturation curves of gene detection number for different sequencing depths (FPKM > 1). Unique mapping reads of different cells have been randomly downsampled to different depths (0.1-0.9 million, 1 million - 8 million), respectively. Only genes with FPKM greater than 1 are selected for the curves. Each line represents a single cell.

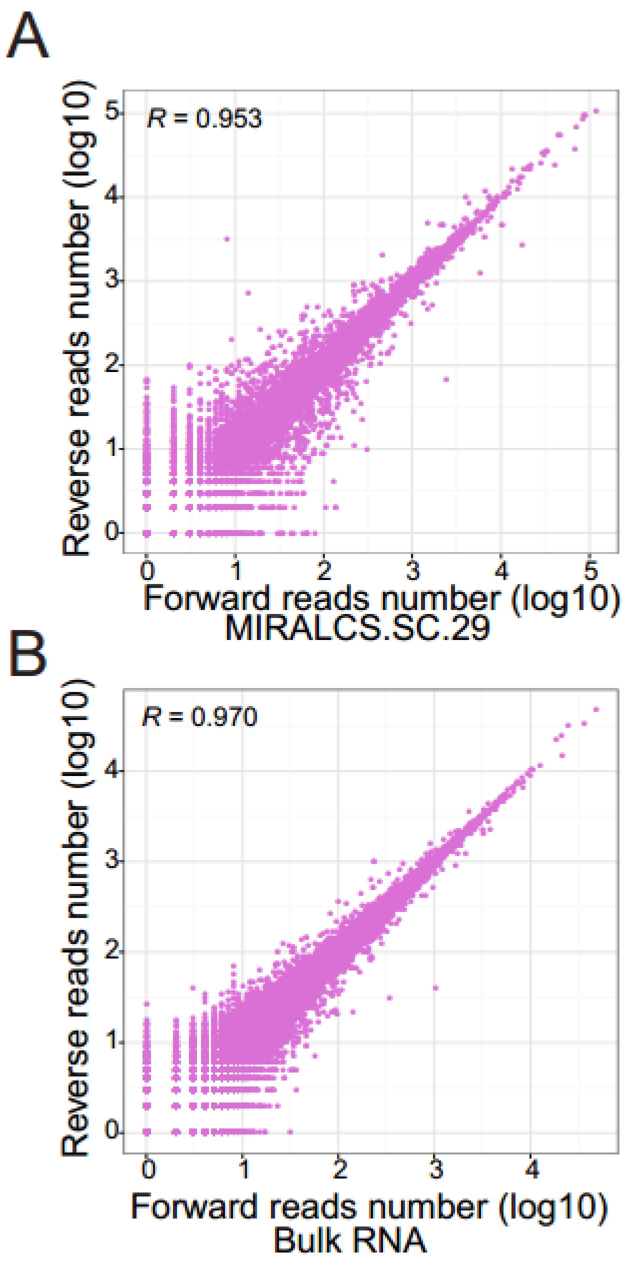

Supplementary Figure 10. Strand bias of the RNA libraries. (A) Strand bias of the single cell libraries. (B) Strand bias of the bulk RNA library. The x coordinate and y coordinate indicate the number of forward reads and reverse reads which were mapped to each gene.

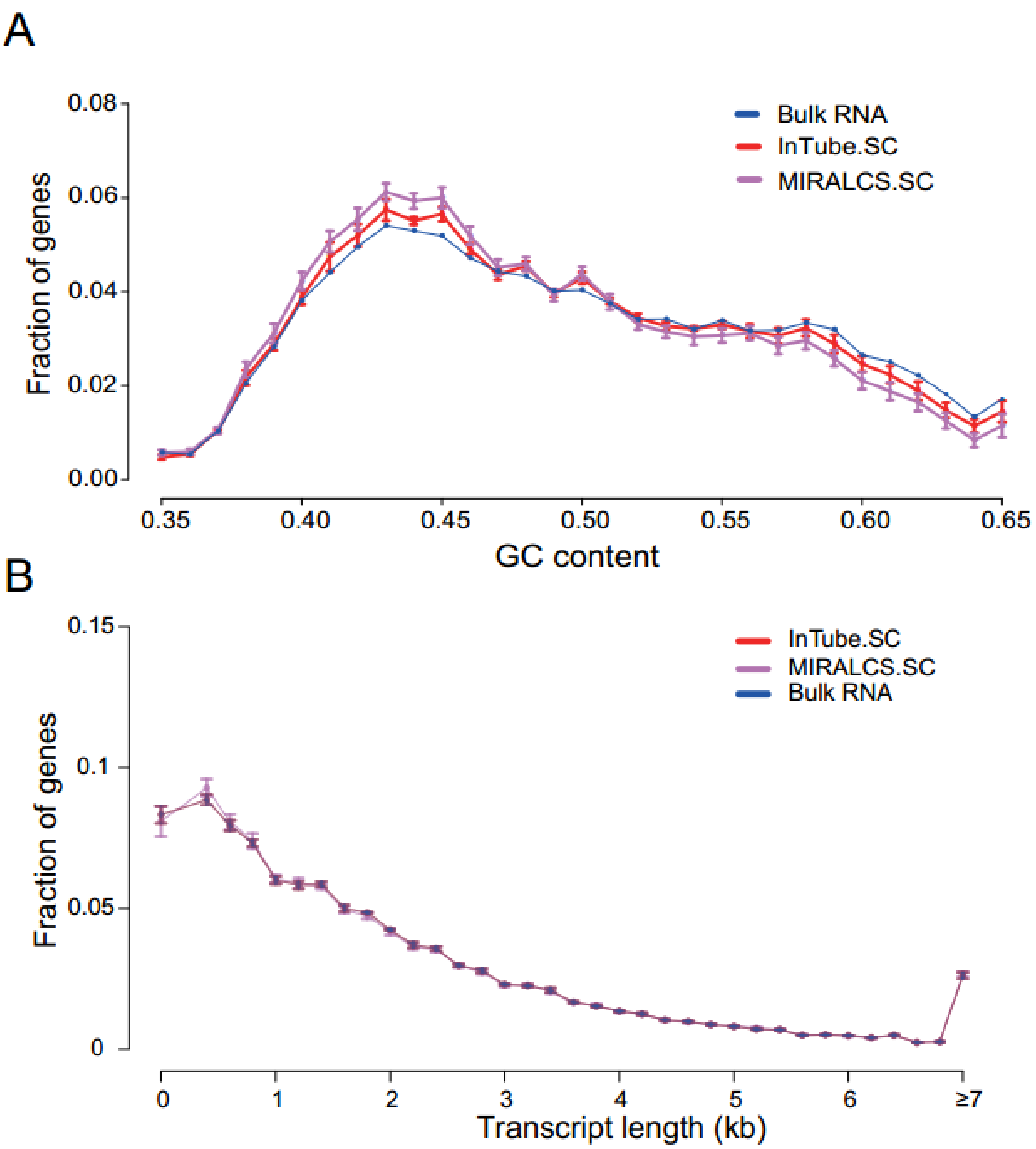

Supplementary Figure 11. The gene distribution by different length of transcripts and GC content. (A) Fraction of genes detected for different transcript lengths. (B) Fraction of genes detected for different CG content.

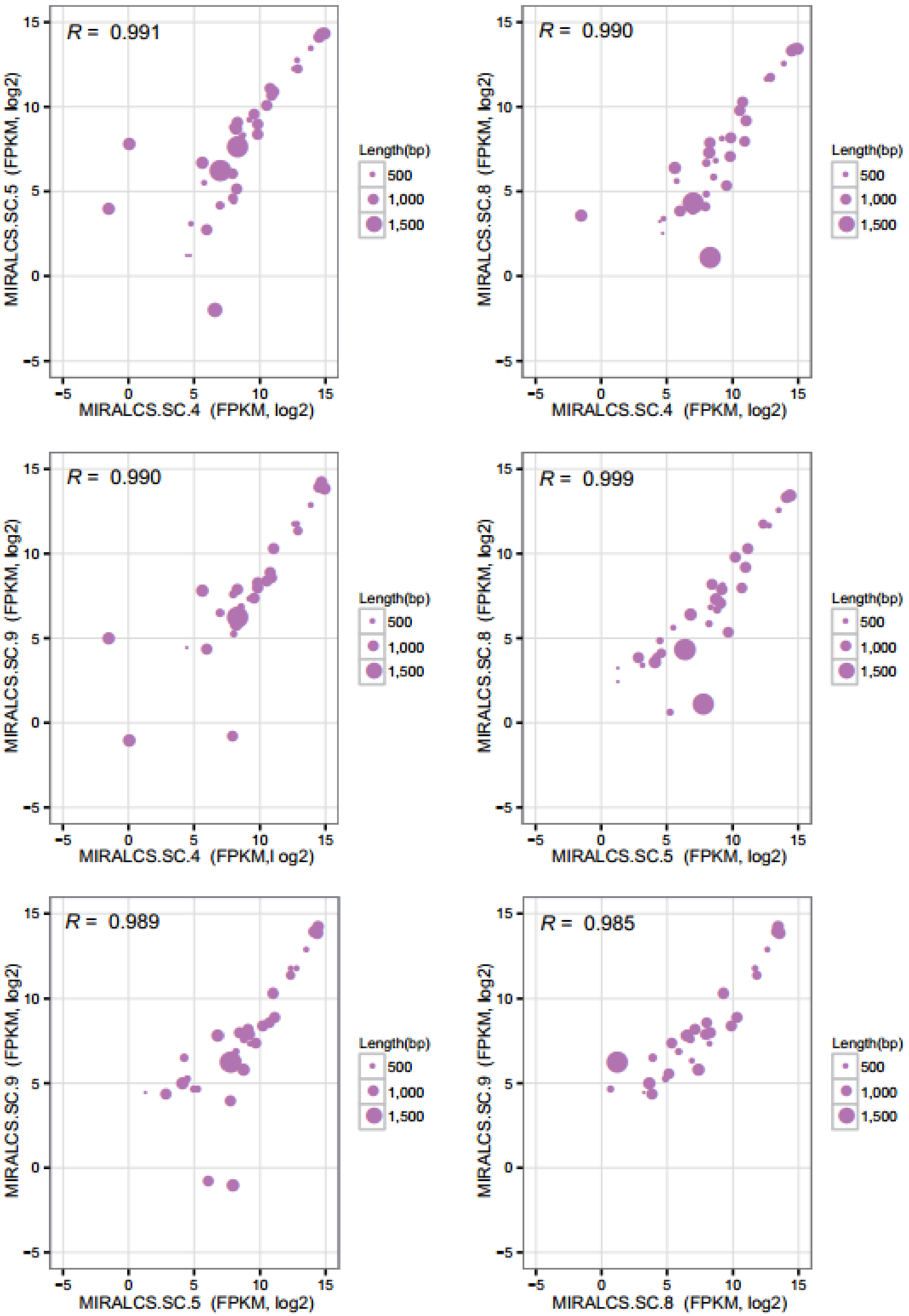

Supplementary Figure 12. The correlation coefficients of pair-wise ERCC spike-ins in different wells. Six results of them are randomly selected.

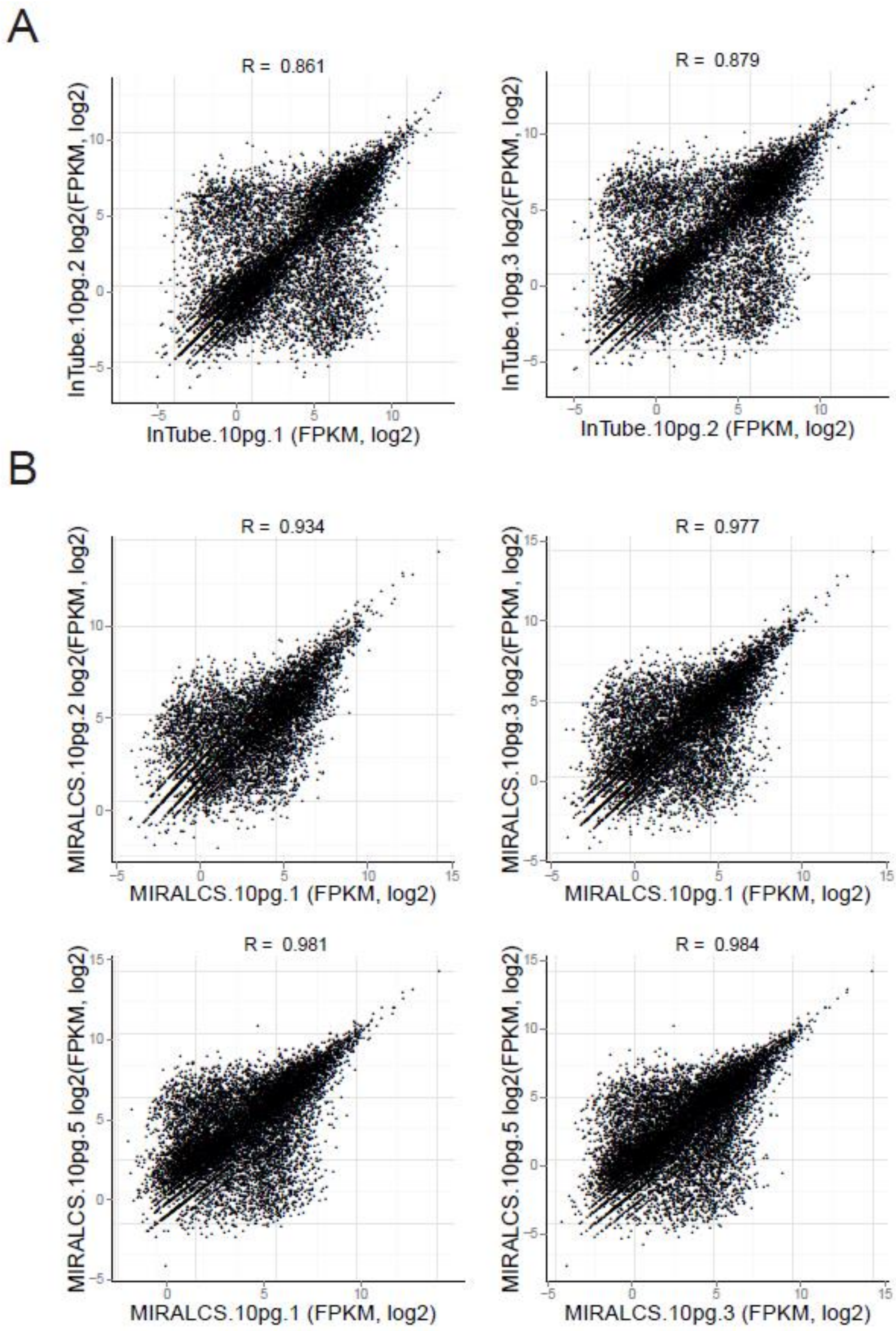

Supplementary Figure 13. The correlation coefficient of pair-wise 10 pg RNA replicates. (A) The pair-wise correlation of tube-based 10pg RNA libraries. (B) The pair-wise correlation of MIRALCS 10pg RNA libraries.

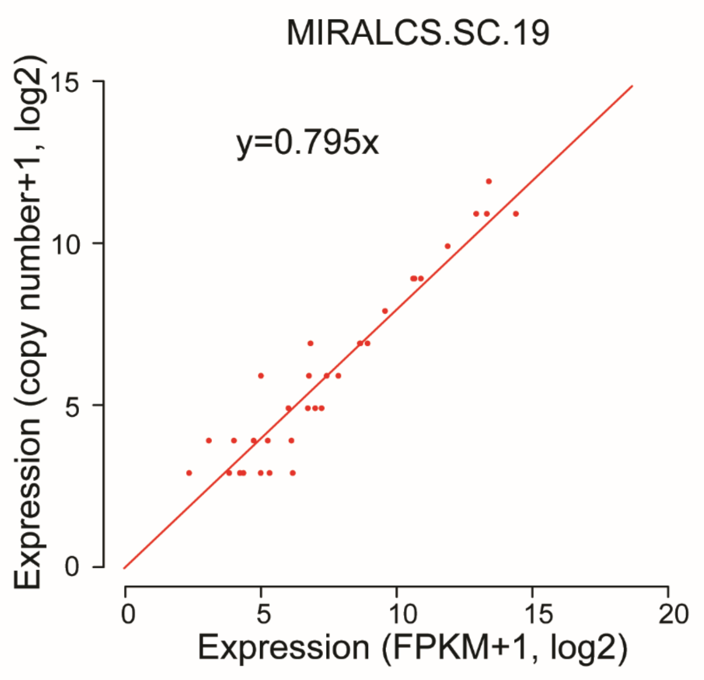

Supplementary Figure 14. The linear regression relationship between FPKM and real mRNA copy number of spike-ins of one randomly selected single cell.

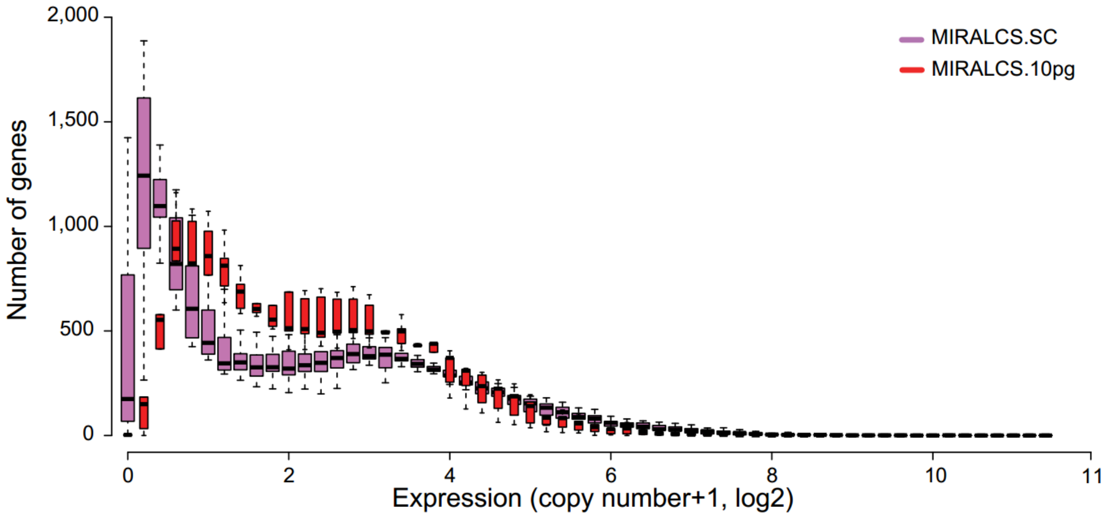

Supplementary Figure 15. Distribution of gene expression levels in estimated copies per cell in single cells and 10 pg RNA replicates.

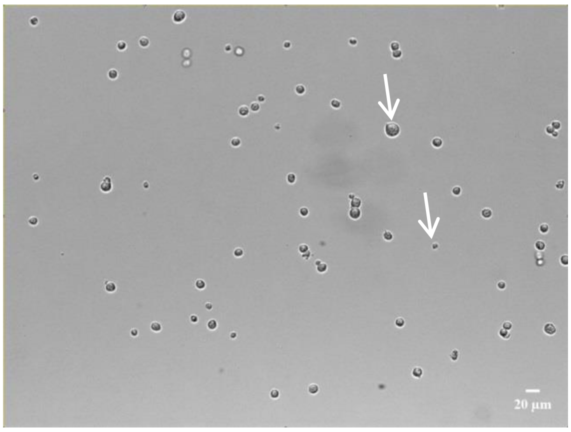

Supplementary Figure 16. The variable sizes of HeLa S3 cells observed by microscope. The white arrows point to typical cells with variable sizes. Scale bar stands for 20 µm.

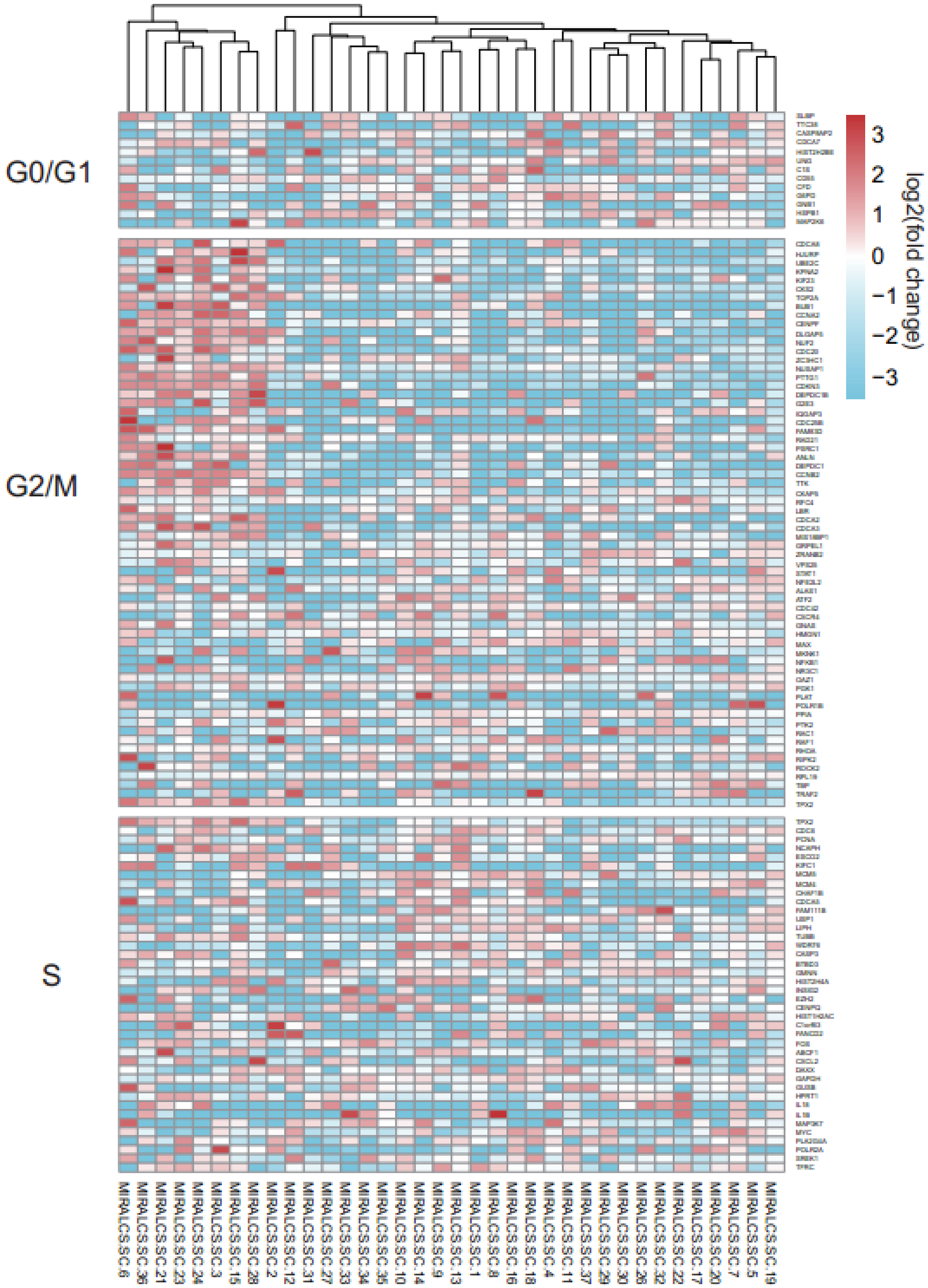

Supplementary Figure 17. Cell clustering according to phase-specific expressed genes. There are 8 cells displaying a higher expression level in G2/M relative genes.

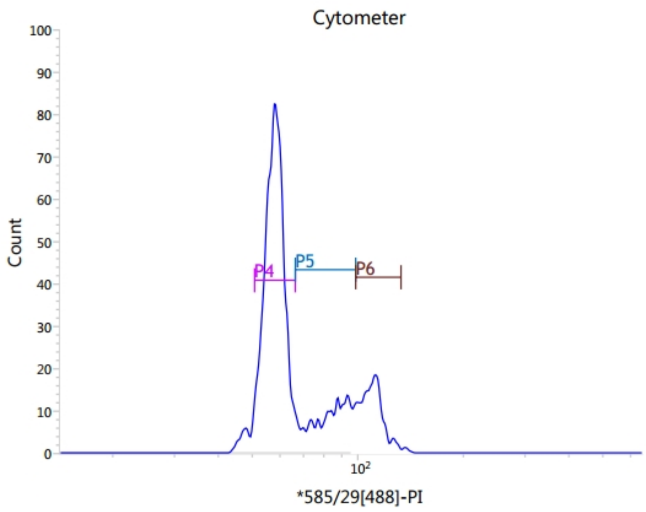

Populations: Cytometer

Populations	Even...	% Total	% Parent
All Events	11,786	100.00%	####
P1	4,270	36.23%	36.23%
P2	3,660	31.05%	85.71%
P3	2,593	22.00%	70.85%
P4	1,678	14.24%	64.71%
P5	520	4.41%	20.05%
P6	431	3.66%	16.62%

Supplementary Figure 18. Cell cycle analysis by flow cytometry. The percentage of G2/M phase (P6) is 16.6%.

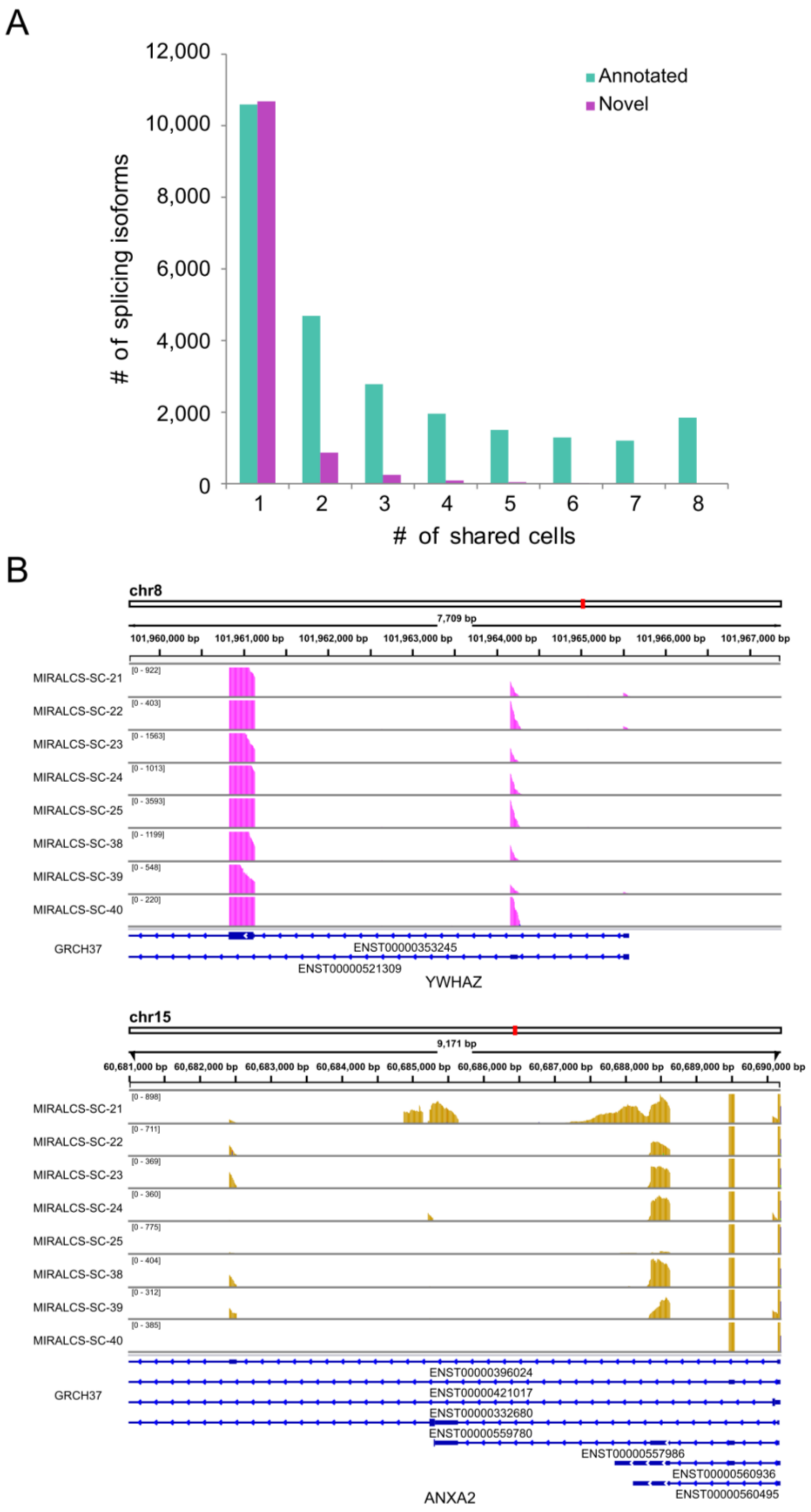

Supplementary Figure 19. Alternative splicing isoform distribution in single cells and reads mapping of gene *AXAN2* and *YWHAZ*. (A) Alternative splicing isoform distribution in single cells. The green bars stand for the number of annotated splicing

isoforms, and the purple bars for the number of novel splicing isoforms. (B) Reads mapping of gene *AXAN2* and *YWHAZ*.

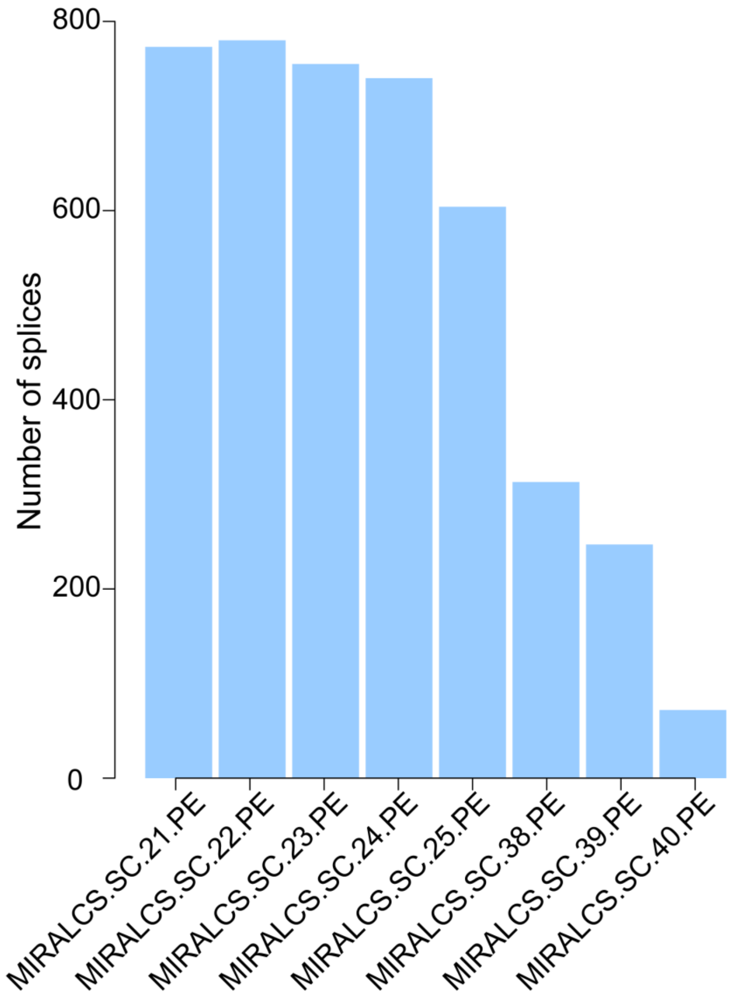

Supplementary Figure 20. The number of detected novel splices in single cells.

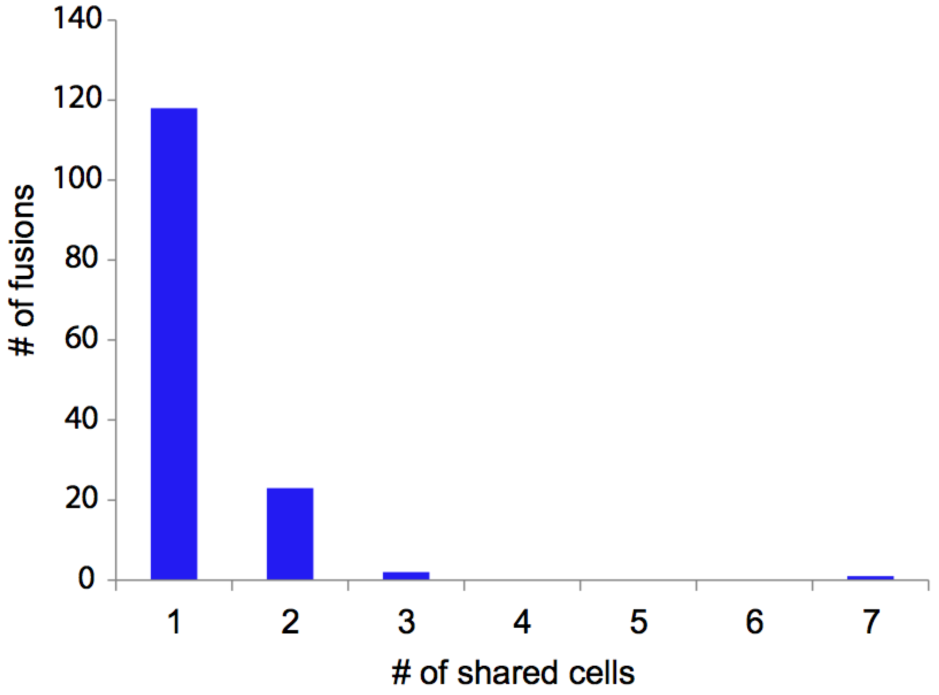

Supplementary Figure 21. The frequency of detected gene fusions in single cells.

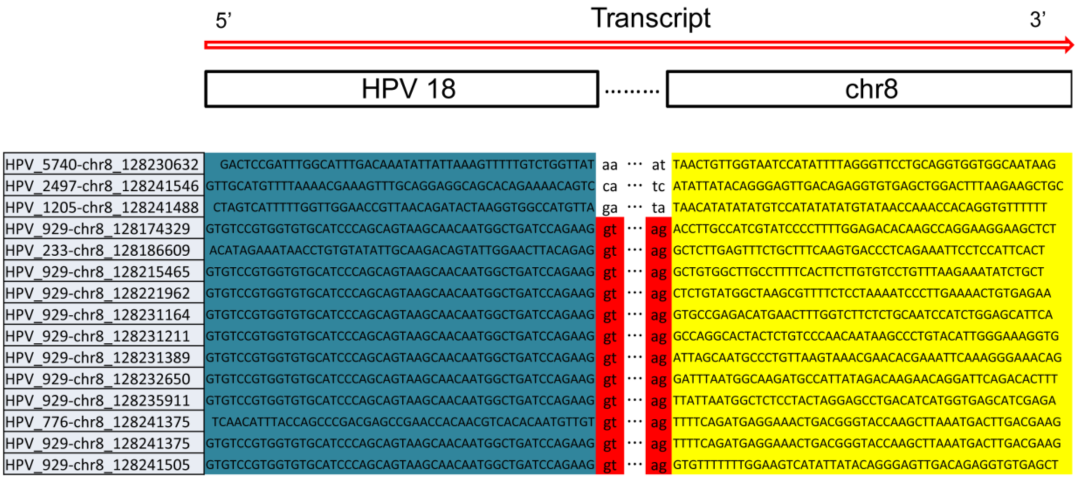

Supplementary Figure 22. Splicing donor-accepter signals detected at the HPV-18/cellular fusion breakpoints.

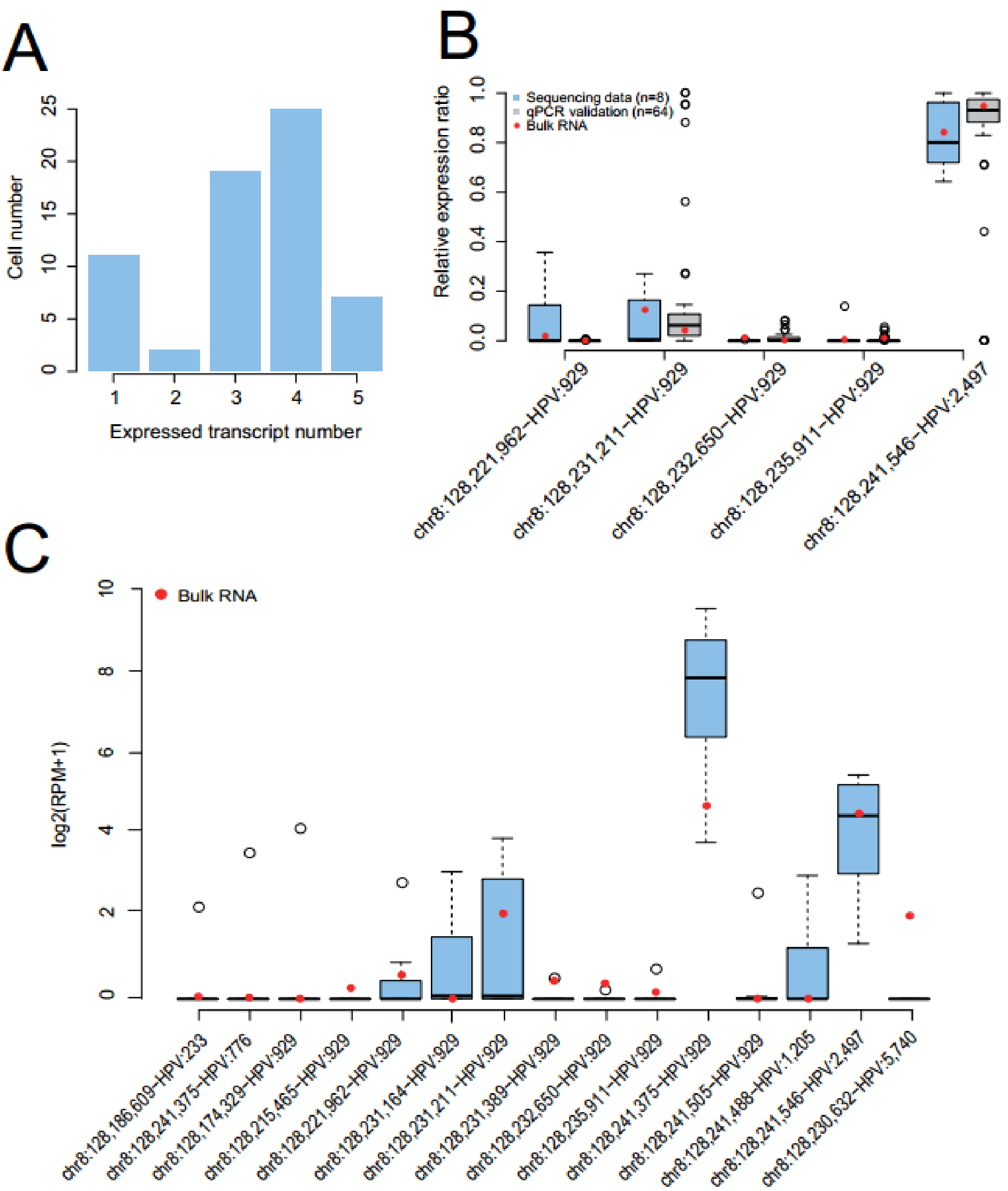

Supplementary Figure 23. Quantification of HPV/human fusions. (A) Distribution of the expressed transcript number of 5 fusions validated in 64 single cells by qPCR. (B) The relative expression ratio of 5 fusions quantified by RNA sequencing data and qPCR validation respectively. (C) The expression abundance of 15 fusions quantified by RNA sequencing data in 8 single cells and bulk RNA. RPM stands for reads per million.

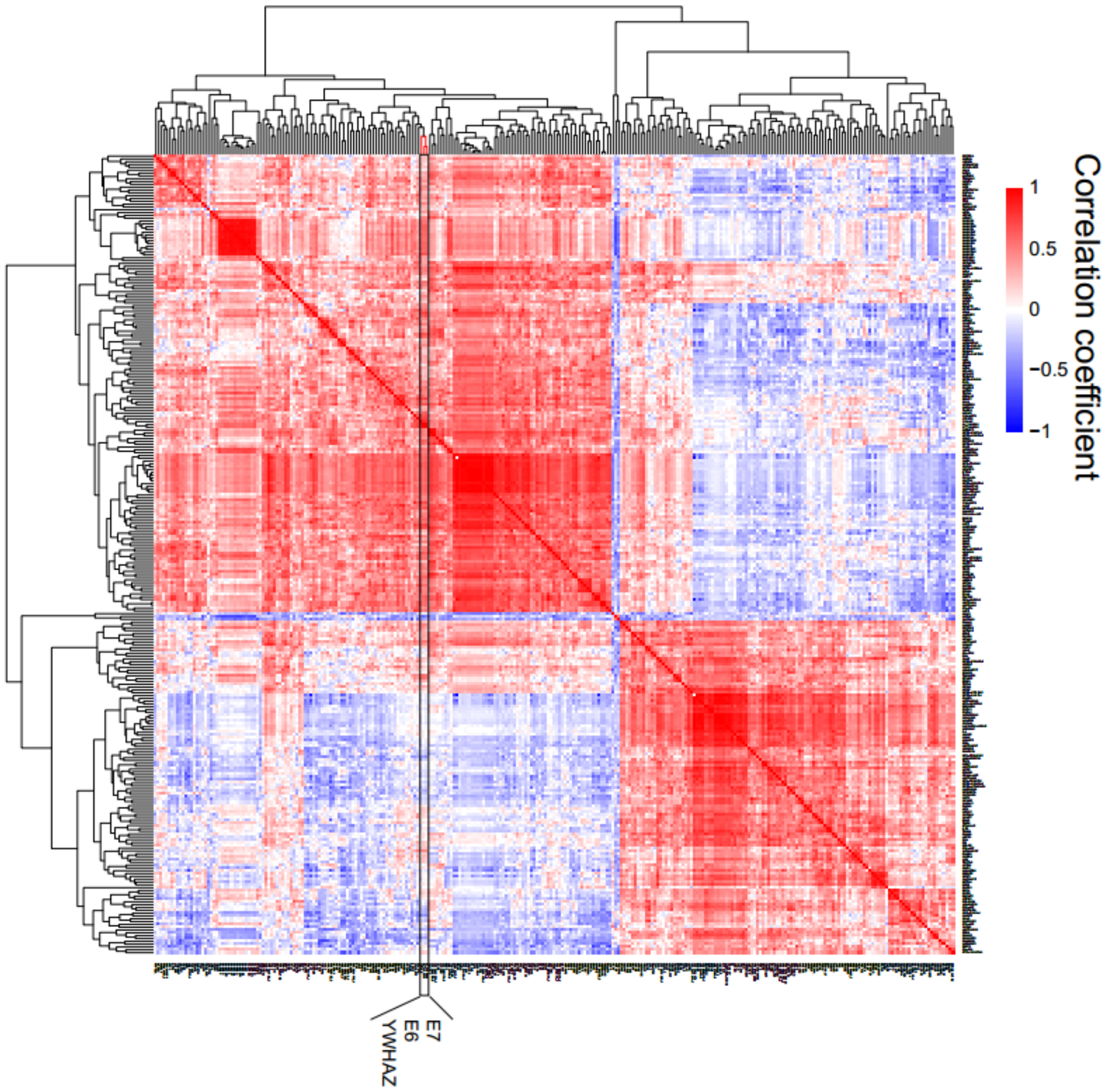

Supplementary Figure 24. Clustering of correlation coefficients of genes in the module which contains *E6* and *E7*.

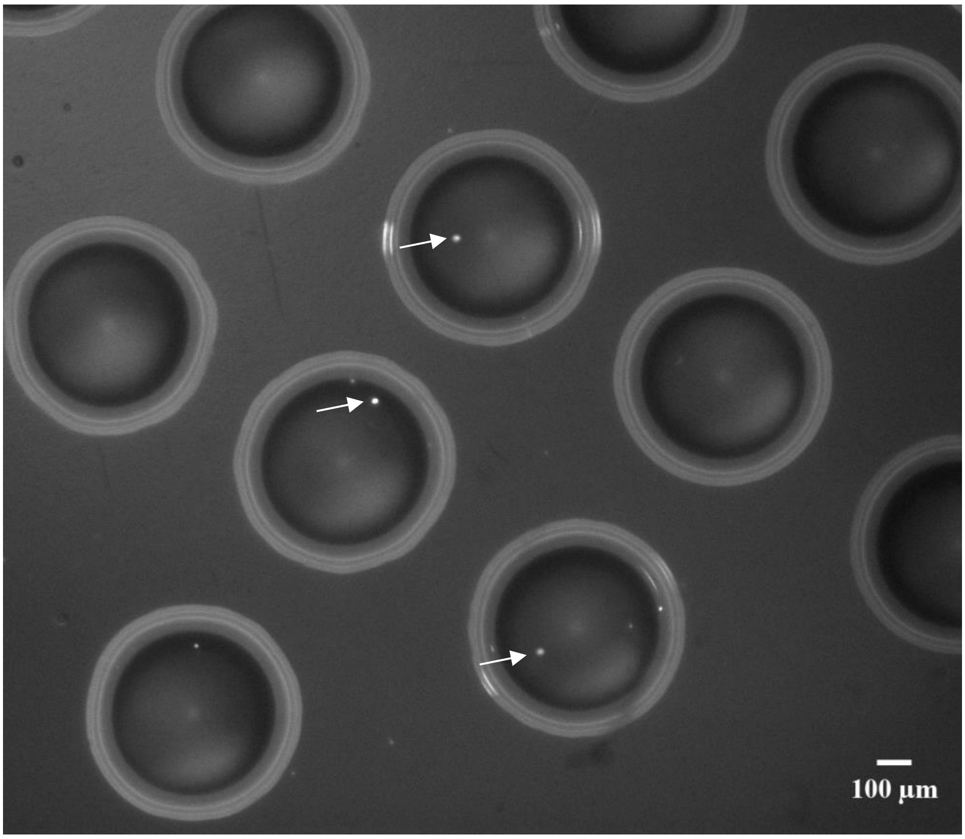

Supplementary Figure 25. Cells dispensed on a plastic film under the microscope. Cell dyed with SYBR green, dispensed on the transparent plastic film and the fluorescence image under the microscope was captured by CCD. The white arrows indicate single cells. Scale bar strand for 100 μm.

Supplementary Table 1. Information of the sample-preparation platform and object (single cell or total RNA) of each library used.

Platform	Object	Sample IDs	Object	Sample IDs
MIRALCS	10 pg total RNA	MIRALCS.10pg.1	Single cell	MIRALCS.SC.13
	10 pg total RNA	MIRALCS.10pg.2	Single cell	MIRALCS.SC.14
	10 pg total RNA	MIRALCS.10pg.3	Single cell	MIRALCS.SC.15
	10 pg total RNA	MIRALCS.10pg.4	Single cell	MIRALCS.SC.16
	10 pg total RNA	MIRALCS.10pg.5	Single cell	MIRALCS.SC.17
	Single cell	MIRALCS.SC.21.PE	Single cell	MIRALCS.SC.18
	Single cell	MIRALCS.SC.22.PE	Single cell	MIRALCS.SC.19
	Single cell	MIRALCS.SC.23.PE	Single cell	MIRALCS.SC.20
	Single cell	MIRALCS.SC.24.PE	Single cell	MIRALCS.SC.21
	Single cell	MIRALCS.SC.25.PE	Single cell	MIRALCS.SC.22
	Single cell	MIRALCS.SC.38.PE	Single cell	MIRALCS.SC.23
	Single cell	MIRALCS.SC.39.PE	Single cell	MIRALCS.SC.24
	Single cell	MIRALCS.SC.40.PE	Single cell	MIRALCS.SC.25
	Single cell	MIRALCS.SC.1	Single cell	MIRALCS.SC.26
	Single cell	MIRALCS.SC.2	Single cell	MIRALCS.SC.27
	Single cell	MIRALCS.SC.3	Single cell	MIRALCS.SC.28
	Single cell	MIRALCS.SC.4	Single cell	MIRALCS.SC.29
	Single cell	MIRALCS.SC.5	Single cell	MIRALCS.SC.30
	Single cell	MIRALCS.SC.6	Single cell	MIRALCS.SC.31
	Single cell	MIRALCS.SC.7	Single cell	MIRALCS.SC.32
	Single cell	MIRALCS.SC.8	Single cell	MIRALCS.SC.33
	Single cell	MIRALCS.SC.9	Single cell	MIRALCS.SC.34
	Single cell	MIRALCS.SC.10	Single cell	MIRALCS.SC.35
	Single cell	MIRALCS.SC.11	Single cell	MIRALCS.SC.36
	Single cell	MIRALCS.SC.12	Single cell	MIRALCS.SC.37
Tube-based	10 pg total RNA	InTube.10pg.1	Single cell	InTube.SC.1
	10 pg total RNA	InTube.10pg.2	Single cell	InTube.SC.2
	10 pg total RNA	InTube.10pg.3	Single cell	InTube.SC.3
	5 ng total RNA	InTube.5ng	Single cell	InTube.SC.4
	5 ng total RNA	InTube.5ng.PE	Single cell	InTube.SC.5

Supplementary Table 2. Information of RNA-seq data.

Sample IDs	Sample accession code	Total number of reads	Number of clean reads (%)	Number of uniquely mapped reads (%)	Read length
MIRALCS.10pg.1	SRS1046095	2,650,640	2,621,015 (98.9)	1,410,668 (53.8)	49
MIRALCS.10pg.2	SRS1046096	2,188,829	2,173,129 (99.3)	354,738 (16.3)	49
MIRALCS.10pg.3	SRS1046097	3,376,802	3,351,539 (99.3)	1,496,316 (44.6)	49
MIRALCS.10pg.4	SRS1046098	4,432,187	4,378,296 (98.8)	1,444,047 (33.0)	49
MIRALCS.10pg.5	SRS1046099	13,688,431	13,626,494 (99.6)	8,513,991 (62.5)	49
MIRALCS.SC.1	SRS1046058	3,325,941	3,315,650 (99.7)	2,787,810 (84.1)	49
MIRALCS.SC.2	SRS1046059	3,864,635	3,850,609 (99.6)	3,428,671 (89.0)	49
MIRALCS.SC.3	SRS1046060	4,978,882	4,961,363 (99.7)	4,200,221 (84.7)	49
MIRALCS.SC.4	SRS1046061	3,833,147	3,821,363 (99.7)	3,305,766 (86.5)	49
MIRALCS.SC.5	SRS1046062	4,672,614	4,658,204 (99.7)	3,946,583 (84.7)	49
MIRALCS.SC.6	SRS1046063	5,117,576	5,099,891 (99.7)	4,362,022 (85.5)	49
MIRALCS.SC.7	SRS1046064	4,962,043	4,944,363 (99.6)	4,056,502 (82.0)	49
MIRALCS.SC.8	SRS1046065	3,328,009	3,318,410 (99.7)	2,630,541 (79.3)	49
MIRALCS.SC.9	SRS1046066	5,536,886	5,520,364 (99.7)	4,704,250 (85.2)	49
MIRALCS.SC.10	SRS1046067	4,107,819	4,094,990 (99.7)	3,281,604 (80.1)	49
MIRALCS.SC.11	SRS1046068	4,102,994	4,090,618 (99.7)	3,427,168 (83.8)	49
MIRALCS.SC.12	SRS1046069	3,544,197	3,532,007 (99.7)	3,020,131 (85.5)	49
MIRALCS.SC.13	SRS1046070	3,068,546	3,059,002 (99.7)	2,409,121 (78.8)	49
MIRALCS.SC.14	SRS1046071	2,451,949	2,445,135 (99.7)	1,908,993 (78.1)	49
MIRALCS.SC.15	SRS1046072	3,372,426	3,362,307	2,827,347	49

			(99.7)	(84.1)	
MIRALCS.SC.16	SRS1046073	4,881,706	4,867,263 (99.7)	4,076,249 (83.7)	49
MIRALCS.SC.17	SRS1046074	4,194,228	4,181,705 (99.7)	3,474,449 (83.1)	49
MIRALCS.SC.18	SRS1046075	3,698,819	3,687,042 (99.7)	3,162,452 (85.8)	49
MIRALCS.SC.19	SRS1046076	3,327,986	3,317,840 (99.7)	2,769,786 (83.5)	49
MIRALCS.SC.20	SRS1046077	4,501,973	4,488,780 (99.7)	3,791,819 (84.5)	49
MIRALCS.SC.21	SRS1046078	10,820,458	10,765,254 (99.5)	9,848,586 (91.5)	49
MIRALCS.SC.22	SRS1046079	10,233,497	10,181,503 (99.5)	9,148,798 (89.9)	49
MIRALCS.SC.23	SRS1046080	9,489,263	9,440,890 (99.5)	8,499,422 (90.0)	49
MIRALCS.SC.24	SRS1046081	11,508,957	11,451,402 (99.5)	10,191,371 (89.0)	49
MIRALCS.SC.25	SRS1046082	12,869,177	12,805,357 (99.5)	11,821,127 (92.3)	49
MIRALCS.SC.26	SRS1046083	6,343,276	6,319,505 (99.6)	5,041,877 (79.8)	49
MIRALCS.SC.27	SRS1046084	6,254,905	6,234,500 (99.7)	4,931,008 (79.1)	49
MIRALCS.SC.28	SRS1046085	5,473,904	5,456,656 (99.7)	4,345,521 (79.6)	49
MIRALCS.SC.29	SRS1046086	9,143,205	9,119,102 (99.7)	7,308,470 (80.1)	49
MIRALCS.SC.30	SRS1046087	4,678,366	4,667,840 (99.8)	3,525,386 (75.5)	49
MIRALCS.SC.31	SRS1046088	6,052,736	6,038,357 (99.8)	2,102,961 (34.8)	49
MIRALCS.SC.32	SRS1046089	8,507,765	8,487,988 (99.8)	6,711,169 (79.1)	49
MIRALCS.SC.33	SRS1046090	10,475,529	10,441,265 (99.7)	7,915,223 (75.8)	49
MIRALCS.SC.34	SRS1046091	16,599,053	16,515,269 (99.5)	11,246,915 (68.1)	49
MIRALCS.SC.35	SRS1046092	12,065,946	11,999,102 (99.5)	9,130,544 (76.1)	49
MIRALCS.SC.36	SRS1046093	14,499,983	14,390,902 (99.3)	11,685,634 (81.2)	49
MIRALCS.SC.37	SRS1046094	11,236,237	11,186,358	8,833,449	49

			(99.6)	(79.0)	
MIRALCS.SC.21.PE	SRS1046101	25,999,028	25,114,733 (96.6)	21,692,166 (86.4)	150
MIRALCS.SC.22.PE	SRS1046102	26,242,289	25,152,679 (95.9)	21,018,293 (83.6)	150
MIRALCS.SC.23.PE	SRS1046103	23,190,348	22,411,711 (96.6)	18,819,969 (84.0)	150
MIRALCS.SC.24.PE	SRS1046104	28,145,215	25,729,561 (91.4)	20,725,957 (80.6)	150
MIRALCS.SC.25.PE	SRS1046105	34,231,992	30,248,920 (88.4)	25,140,263 (83.1)	150
MIRALCS.SC.38.PE	SRS1046106	25,394,834	24,386,456 (96.0)	20,484,030 (84.0)	150
MIRALCS.SC.39.PE	SRS1046107	28,084,700	26,850,835 (95.6)	18,848,217 (70.2)	150
MIRALCS.SC.40.PE	SRS1046108	23,129,962	22,206,277 (96.0)	19,092,129 (86.0)	150
InTube.5ng	SRS1046049	8,225,060	8,207,225 (99.8)	6,212,557 (75.7)	49
InTube.10pg.1	SRS1046055	8,973,138	8,964,541 (99.9)	6,149,736 (68.6)	49
InTube.10pg.2	SRS1046056	9,004,201	8,995,705 (99.9)	5,322,883 (59.2)	49
InTube.10pg.3	SRS1046057	13,284,347	13,272,330 (99.9)	6,690,585 (50.4)	49
InTube.SC.1	SRS1046050	4,374,029	4,359,679 (99.7)	3,868,664 (88.7)	49
InTube.SC.2	SRS1046051	4,536,723	4,522,586 (99.7)	3,739,420 (82.7)	49
InTube.SC.3	SRS1046052	5,033,844	5,019,666 (99.7)	1,723,724 (34.3)	49
InTube.SC.4	SRS1046053	4,587,821	4,573,315 (99.7)	4,085,848 (89.3)	49
InTube.SC.5	SRS1046054	4,309,279	4,296,639 (99.7)	3,750,466 (87.3)	49
InTube.5ng.PE	SRS1046100	46,307,229	41,923,767 (90.5)	29,673,490 (70.8)	90

Supplementary Table 3. Cell settling rate in Percoll solution of different concentrations.

Component	Cell concentration (cells/10μl)	Cell concentration 30min later (cells/10μl)	Remaining
PBS	145	10	6.90%
10% Percoll solution	105	35	33%
20% Percoll solution	85	36	42.30%
20% Percoll solution	38	34	89.50%
20% Percoll solution	26	24	92.30%

Supplementary Table 4. The Ct and Tm values of samples used to validate the selection mechanism.

Sample	Ct	Tm	2100 result
negative control	15.9	79.7	negative
negative control	16.3	79.9	negative
negative control	16.5	79.8	negative
negative control	16.8	79.8	negative
negative control	16.9	79.8	negative
predicted non-target well	15.4	79.6	negative
predicted non-target well	15.4	87.2	negative
predicted non-target well	15.4	87.2	negative
predicted non-target well	15.5	79.6	negative
predicted non-target well	16.2	87.4	negative
predicted non-target well	16.2	87.5	negative
predicted non-target well	16.0	86.0	negative
predicted non-target well	15.4	78.5	negative
predicted non-target well	15.5	78.9	negative
predicted non-target well	15.6	79.4	negative
predicted non-target well	16.2	87.4	negative
predicted non-target well	16.2	79.0	negative
predicted non-target well	16.3	79.2	negative
predicted non-target well	16.4	78.8	negative
predicted non-target well	16.4	85.5	negative
predicted non-target well	16.5	85.5	negative
predicted non-target well	16.8	78.9	negative
predicted non-target well	16.8	85.9265	negative
predicted non-target well	16.9	79.12568	negative
predicted non-target well	17.1	85.50832	negative
positive control	12.8	86.00924	positive
positive control	12.9	85.58861	positive
positive control	12.9	86.06567	positive
positive control	13.0	85.49609	positive
positive control	13.0	86.09607	positive
predicted target well	13.0	88.97237	positive
predicted target well	13.0	88.06715	positive
predicted target well	13.0	88.06403	positive
predicted target well	12.7	87.58961	positive
predicted target well	12.8	85.35646	positive
predicted target well	12.9	88.46877	positive
predicted target well	13.0	87.9	positive
predicted target well	12.9	88.4	positive
predicted target well	13.0	87.9	positive
predicted target well	13.2	86.7	positive

predicted target well	13.1	88.6	positive
predicted target well	12.7	85.7	positive
predicted target well	12.4	88.2	positive
predicted target well	12.4	87.6	positive
predicted target well	12.9	88.8	positive
predicted target well	13.1	88.6	positive
predicted target well	12.9	88.2	positive
predicted target well	12.6	87.9	positive
predicted target well	13.1	87.4	positive
predicted target well	13.1	87.4	positive

Supplementary Table 11 Reads support by HeLa S3 cell line genome sequencing

Human chromosome and position	HPV integration site	Mate pair reads	Clipped reads
chr8:128,232,632*	HPV:5,739	7	0
chr8:128,233,367	HPV:3,088	3	0
chr8:128,234,255	HPV:7,883	12	6
chr8:128,241,545	HPV:2,496	1	13

*This pair of breakpoint is shared by the other 3 pairs of breakpoints.

Supplementary Table 13 The comparison between MIRALCS and Fluidigm C1 system.

	MIRALCS	Fluidigm C1 system
Throughput	> 500 single cells	96 single cells
Full-length mRNA	Yes	Yes
Reaction scale	nL	nL
Time consumption	~6 hours	~4 hours
Data type	Hiseq 2000 Singled-end 50 bp	Hiseq 4000 Paired-end 151 bp
Mapped reads rate	~75% (N = 37)	~60% (N = 220)
Detected gene number*	7,654 (3,854~10,015)	5,619 (1,499~8,585)

*To avoid the influence of sequencing depth, we only selected the single-cells with more than 1.5Mreads, and detected gene number only calculate the genes with FPKM > 1.

Supplementary Note 1 Operation steps of MIRALCS.

All reagents adding of MIRALCS is finished by using MSND, and the amplified cDNA was prepared by MIRALCS following the steps below. Samples and 20 μl of reagents, including lysis buffer, reverse transcription mix and PCR-mix, were pre-added into 36 wells of a 384-well plate, and then dispensed into the micro-well chip in order. The chip was centrifuged after each addition for 5 minutes under 2600 rcf. As a proof-of-concept experiment, 20% Percoll solution was used as negative control and HeLa S3 total RNA with the concentration of 1, 10, 40 and 160 pg/50 nl was used as positive control to assess the feasibility of the protocol. The reverse transcription was performed following the Smart-seq2 protocol with slight modifications. The flow chart and the reagent list are shown below.

Stage 1 Lysis buffer loading. Lysis buffer is prepared as follow, then they are added into 36/384 well plate. ERCC RNA spike in was purchased from Ambion, Life Technologies.

Supplementary Table 14. The components of lysis buffer.

Component	Volume in each well (nl)	Total volume (μl)
10%Triton X-100	0.5	7.56
40 U/μl RNase Inhibitor	1.25	18.9
10 μM Oligo-dT Primer	12.5	189
10 mM dNTP	12.5	189
Spike in (1 : 116250)	23.25	351.54
Total	50	756

Stage 2 Sample loading and lysis. Cell suspended in 20% Percoll solution with a concentration of 2~8 cells/μl, negative control of 20% Percoll solution without cells and positive control of total RNA with concentration of ~10 pg/well are added into 36 wells of 384-well plate, and then dispensed into the micro-well chip. After sample loading, micro-well chip is incubated at 72 ℃ for 3 min and 4 ℃ for 5min for the cell lysis.

Stage 3 Reverse transcription reaction. Reverse transcription reagents are prepared following the table below. The mixed reagents are added into 36 wells of 384-well, and then dispensed into the micro-well chip. After reverse transcription mix dispensing, the micro-well chip is incubated at 42 ℃ for 90 min, 2 cycles of 50 ℃ for 2 min and 42 ℃ for 2 min, and then incubated at 70 ℃ for 15 min, 12 ℃ for 5 min for the mRNA reverse transcription.

Supplementary Table 15. The components of reverse transcription reaction buffer.

Component	Volume per well (nl)	Total volume (μl)
200 U/μl SSII	6	90.72
5×SuperScript II	16	241.92

First-Strand Buffer		
5 M Betaine	16	241.92
100 mM $MgCl_2$	7.2	108.86
100 mM DTT	2.	30.24
100 µM TSO	0.8	12.1
40 U/µl RNase inhibitor	2	30.24
Total	50	756

Stage 4 The cDNA Amplification. The PCR buffer is prepared by following the table below, added into 36 wells of a 384-well plate, and then dispensed into the micro-well chip. After PCR buffer dispensing, the micro-well chip is incubated in Smart cycling thermal instrument at 98 ℃ for 3 min，22 cycles of 98 ℃ for 15 s，67 ℃ for 20 s and 72 ℃ for 6 min，and then 72 ℃ for 5min, 72 ℃ to 98 ℃, 0.4 ℃ per step, 98 ℃ for 15 s, 67 ℃ for 20 s, 72 ℃ for 6 min,15 ℃ for 5 min.

Supplementary Table 16. The components of cDNA amplification reaction buffer.

Component	Volume per well (nl)	Total volume (µl)
2×KAPA HiFi HotStart ReadyMix	41.67	630
10 µM IS PCR Primer	0.83	12.6
Nuclease-free water	5	75.6
SYBR Green I (20×)	2.5	37.8
Total	50	756

Based on the Ct and Tm values produced in stage 4, we selected the target-wells, and output their position on the chip. Then after inputting the table of position of target wells to the software of the automatic extractor, we transported the amplified cDNA products of single cells from chip to 96-well plate and stored at -20 ℃.
